# A Comparison Study on Traditional Mixtures of Herbal Teas Used in Eastern Mediterranean Area

**DOI:** 10.3389/fphar.2021.632692

**Published:** 2021-04-23

**Authors:** Concepción Obón, Diego Rivera, Elena Fonollá, Francisco Alcaraz, Latifa Attieh

**Affiliations:** ^1^ Departamento de Biología Aplicada, EPSO, Universidad Miguel Hernández de Elche, Orihuela, Spain; ^2^ Departamento de Biología Vegetal, Facultad de Medicina, Universidad de Murcia, Murcia, Spain; ^3^ International School of Business (ISB), Modern University for Business and Science (MUBS), Beirut, Lebanon

**Keywords:** ethnobotany, ethnopharmacology, pharmacognosy, unani, traditional medicine, phytotherapy, pharmacy

## Abstract

Multipurpose herbal teas with numerous ingredients, in which flowers are the main component, are common in the traditional medicine and pharmacy of Greece and the Eastern Mediterranean countries. In this study, we combine ethnobotany and ethnopharmacology field work techniques and botany and pharmacognosy laboratory methods for the study of traditional herbal mixtures with flowers, we identify their botanical ingredients and record the local medicinal uses of these mixtures, in Greece, Lebanon, Syria, Iran and Turkey. These, and their industrial versions, are analyzed, using morphological and multivariate analysis techniques in order to determine marker species, relevant patterns of combination and local styles. The medicinal properties attributed to the different flowers are discussed in relation with their role in the mixtures. These blends are consumed for their relaxing, digestive, and anti-infective properties. These mixtures are not consumed as a treatment when one is sick but rather to avoid getting sick, as a preventive measure. The formulations can reach forty ingredients (*sarantha* in Greek, *arbain* in Arabic language of Palestine), usually entire or coarsely chopped in the more traditional formulations, leading to extreme variability of individual doses. We ask what biological signification this randomness can have. To give an answer requires new and more comprehensive pharmacological approaches. The flowers of Rosaceae, Asteraceae, Lamiaceae, Malvaceae and Fabaceae species characterize these mixtures in which other materials (roots, leaves, and fruits) and other species are present as well. Flowers of some species, particularly of Fabaceae, are exclusively used in mixtures, and their use in monospecific herbal teas is not yet recorded. We draw attention on the urgent need in exhaustively recording in Greece and the Near East, the formulation and use of traditional herbal mixtures and their numerous local variants. To consider these mixtures and the contribution of flowers (most mixtures receive the general name of tea of flowers) merits further extensive study.

## Introduction

Typical angiosperm flowers appeared more than 150 million years ago in the evolutionary history of plants, early in a pre-Cretaceous period, associated with pollinating insects that played a decisive role in the origin and early evolution of the angiosperms (Ren, 1998). Most plant–pollinator relationships are considered to be mutually beneficial as plants derive reproductive benefits (pollen export and deposition, fertilization) in exchange for resources (nectar, pollen, oils) that directly or indirectly enhance the pollinator’s fitness (Raguso, 2004). In this context, floral scent functions and visual cues attract pollinators, induce them to land, indicate a reward’s presence and location, and teach pollinators to associate the reward with specific flowers (Raguso, 2004). The chemicals that are involved in this ensemble of relationships stand available in the flowers in a wide repertory including: volatile compounds responsible of their scents (fatty acid derivatives, benzenoids, phenylpropanoids, isoprenoids, nitrogen containing compounds, etc.) (Jürgens et al., 2003; Raguso 2004; Dötterl & Vereecken, 2010); pigments coloring the flowers such as anthocyanins (Saito and Harborne 1992), flavonoids and betalains (Miller et al., 2011); nectars with amino acids, carbohydrates, vitamins, mineral ions and others (Morris et al., 2020); and finally toxic substances such as alkaloids, non-protein aminoacids, lectins, ammonia, and heavy metals (Adler, 2000).

Such a wide repertoire of biologically active compounds attracted the attention not only of bees and other pollinators, but also of hominids, who used the flowers of numerous species for food and medicine. The presence of large amounts of pollen from medicinal plant species at the burial sites of the Neanderthal Shanidar’s Cave, is interpreted to mean their early medicinal use (Lietava, 1992). Flowers such as artichokes, capers, pumpkin flowers, roses, violets, cauliflower and broccoli are usually eaten as food and are considered as a primary source of nutrients (notably carbohydrates and vitamins). Interestingly, some of them are also included in the materia medica on account of their contents in bioactive compounds (Inocencio et al., 2000; Rivera and Obón, 2004; Fernandes et al., 2017).

The relevance of flowers in the traditional medicine of the Eastern Mediterranean area, mainly in the Balkans and West Asia is indicated by classical authors such as Theophrastus, Hippocrates and Dioscorides (Rivera et al., 2012). Notably Dioscorides, 1st century AD, mentions *Krokos* (saffron), *Rhodinon* (oil of rose petals), *Susinon* (lilies’ oil), *Narcissinon* (oil of narcissus flowers), *Iasmelaion* (oil of Persian jasmine), *Kystos arren* (Cretan rockrose flowers), *Rhodon* (scented roses), *Kytinoi* (calyx of pomegranate flowers) and *Balaustion* (flowers of wild pomegranate), *Leukoion* (?), *Buphthalmon* (a type of chamomile), *Chrysanthemon* (crown marigold), *Ageraton* (milfoil), *Erigeron* (groundsel), *Aster Attikos* (sea aster), *Ion* (violet) and others (Osbaldeston and Wood, 2020). Most appear illustrated in the Anicia Juliana codex of Wien (Mazal, 1998).

Teas of flowers, or with flowers mixed with other ingredients, are common in the traditional medicine of the Balkans, West Asia and East Mediterranean area under different names such as “*zhourat*” (flowers) (Carmona et al., 2005; Obón et al., 2014) or “*shai alwird*” (Damask rose tea). Other rose species and cultivars (Baser et al., 1986; Vinokur el al. 2006; Baser et al., 2013), jasmine, *Hibiscus sabdariffa* (Saeed et al., 2013), chamomile (Guzelmeric et al., 2017) and other species are also used in traditional herbal teas.

Numerous medicinal single-species floral teas were recorded in Hatay Province (Turkey), including Antakya and Defne, by Güzel et al., (2015) and Güzel and Guzelsemme, (2018), with *Achillea setacea* Waldst & Kit., *Alcea setosa* (Boiss.) Alef., *Calicotome villosa* (Poir.) Link, *Cistus creticus* L., *Cota palaestina* Kotschy, *Eleagnos angustifolia* L., *Erica manipuliflora* Salisb., *Helichrysum sanguineum* (L.) Kostel., *H. plicatum* DC., and *H. stoechas* (L.) Moench, *Hibiscus sabdariffa* L., *Lavandula stoechas* L., *Malva punctata* (All.) Alef., *Matricaria chamomilla* L., *Punica granatum* L., *Rosa* x*damascena* Mill., *Sambucus nigra* L., *Sideritis huber-morathii* Greuter & Burdet, *S. libanotica* Labill., *S. perfoliata* L., *S. syriaca* subsp. *nusairiensis* (Post) Hub.-Mor. or *Viola odorata* L., among others, but no information on mixtures. Although to a lesser extent, Akgül et al. (2016) also recorded single-species floral teas in Cappadocia (Turkey), Akgul et al. (2018) in Mardin Province (Turkey), Güneş et al. (2017) in Adana Province (Turkey) and Sargin et al. (2015) in Manisa (Turkey).

Herbal mixtures constitute an important chapter in different lines of ethnopharmacological research and represent a large volume of information. The relevance of these mixtures in folk medicine could be explained as a response to diseases of multicausal etiology or by a possible multipurpose effect of the mixture as opposed to the effect of each individual ingredient (Gras et al., 2018). Numerous herbal blends have been used for centuries and have been shown to improve health. The study of these mixtures is very complex but, at the same time, promising. Knowledge of the composition of these traditional blends can help develop new blends that can improve the health of citizens (Ndhlala et al., 2011; Barros et al., 2012; Obón et al., 2014).

They are usually made in a traditional way at home or by herbalists in their stores, using whole or slightly fragmented ingredients. However, there is a growing trend toward their transformation into industrial products, either in the form of labeled bags containing the mixture of ingredients or in individual bags in which the ingredients are reduced to powder. On the other hand, ethnopharmacological studies in the area have focused on analyzing isolated ingredients that are sold in markets or in traditional herbal shops, while few studies have been dedicated to mixtures. Tsioutsiou et al. (2019) in their study on medicinal plants used in Central Macedonia (Greece) describe the combinations when reporting on each individual plant.

Our interest focused on the study of complex mixtures that contain a remarkable fraction of flowers, whatever their primary use was. These involve “*zhourat*” in Syria and Lebanon, “*sarantha*” in different parts of Greece and “*karteraki*” in Crete (Greece), “tea of flowers” and “*osmanlı çayı*, Ottoman tea” in Turkey, and “*demonush*”, “*dammnusch*” or “*dammnous*” in Iran. We also included the industrial versions of these when available.

This study aims at determining the relevance of flowers in herbal mixtures that are sold and consumed in the Eastern Mediterranean region and their possible contribution to the medicinal properties that the informants attribute to each of the mixtures, and determining the impact of industrial processing in the evolution of these traditional formulations. This is done by analyzing representative samples from traditional and industrial sources and reviewing relevant phytochemical and pharmacological evidence. Other objectives of the study include identifying the prominent species and families and how different species appear associated in their mixtures, analyzing the existence of geographical patterns and cultural styles, and finally discussing their main contribution and risks.

## Materials and Methods

### Information on Uses

Interviews were conducted with a total of 25 farmers, housewives, shepherds and herbalists from Lebanon, 10 herbalists in Greece (Naxos, Crete, Santorini and mainland), three herbalists in Damascus (Syria) and one herbalist each in Turkey and Iran. The study’s informants were asked to comment on health benefits of these herbal teas if any. For the packed industrial commercial versions we consulted the information in the labels of the product and those displayed in the different webpages of the brands. The indications recorded from our informants or from the labels concerning the therapeutic indications attributed to each mixture are summarized in Supplementary Table S1.

Our informants were informed of the purpose of our study and prior consent for use in scientific papers of the information was obtained. Herbalists were selected among those available in the locality on reason of the complexity of the mixtures they prepared and the presence of flowers in these. The rest of informants were selected based on their local reputation of knowing how to prepare traditional herbal tea mixtures.

### Plant Material

The study includes 93 samples. These are 83, different samples of herbal mixtures and 10, a comparison repertory of complex spices mixtures, which are usual in the Mediterranean area and West Asia (Details are available in Supplementary Table S1). The methodology concentrates in the analysis of herbal ingredients (flowers, leaves, fruits, barks and others, not chemical compounds). It involves the botanical identification of these, based of macro and micromorphological characters of the materials.

**FIGURE 1:**
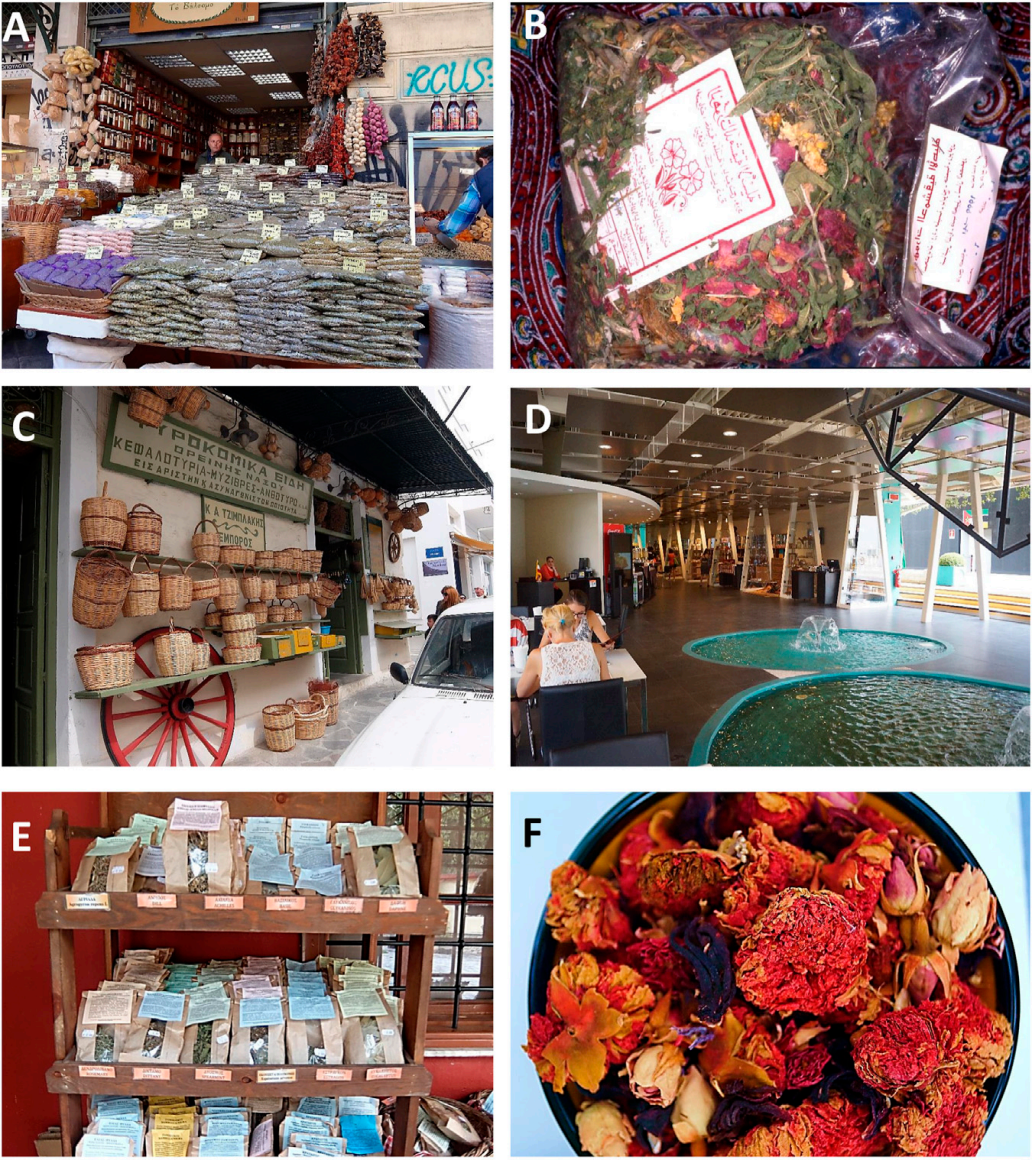
Herbal teas with flowers from Greece and the Eastern Mediterranean. **(A)** *Βαρβάκειος Αγορά, Varvakeion* Market in Athens, Herb Shop on Athinas Street; **(B)** “*Zhourat*” bag from the Damascus market; **(C)** Grocery store in Naxos on *Sokratous Papavasiliou* Street; **(D)** Shop with herbal teas of the Iranian Pavillion at Expo 2015, Milan; **(E)** Mixtures of medicinal plants from the monastery of *Agios Dionisos* (Lithohoro), located at the foot of Olympus; **(F)** Ottoman Style herbal tea with: carcade, Damask rose buds and pomegranate flowers. Photos: C. Obón and D. Rivera.

**FIGURE 2:**
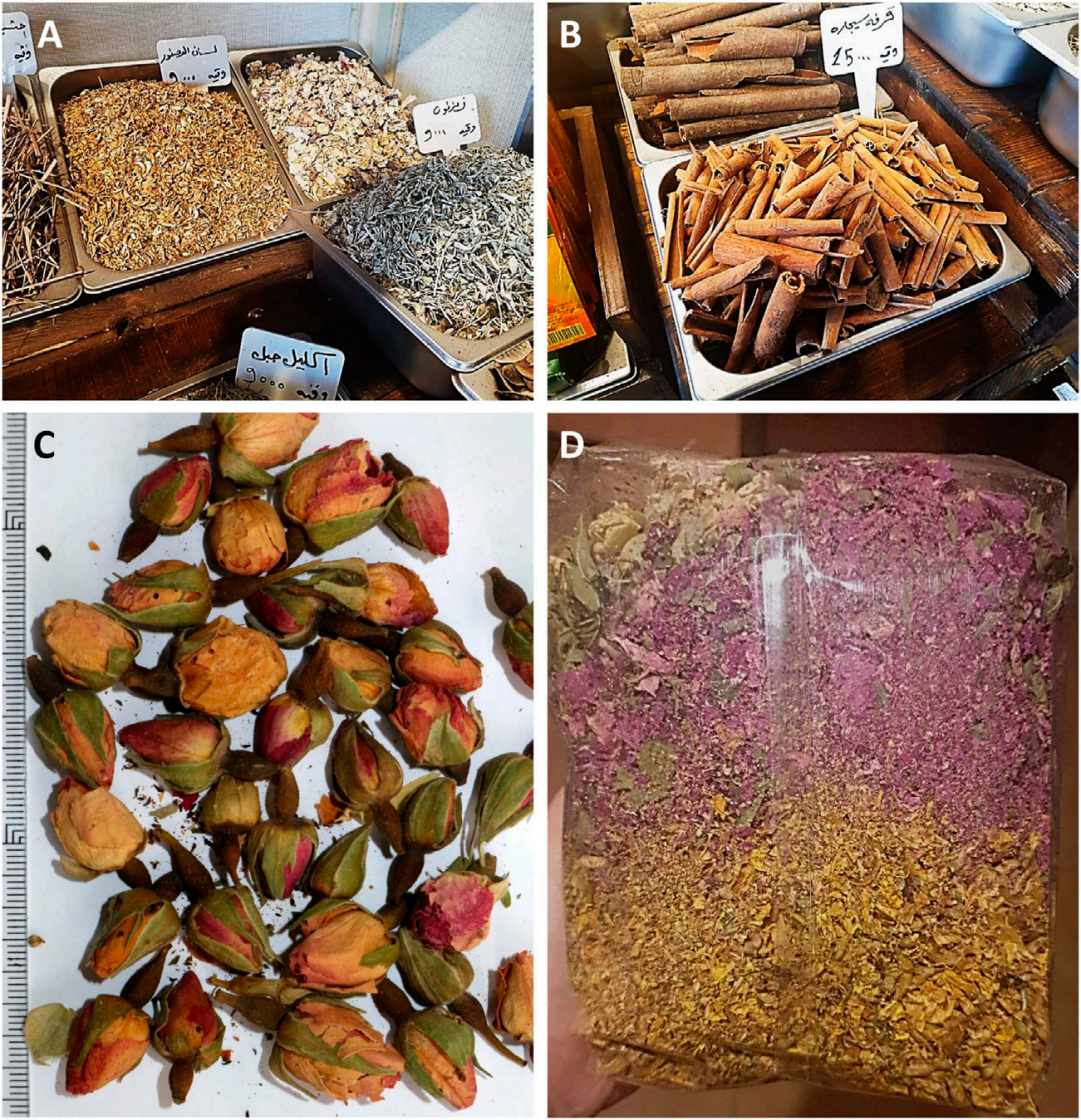
Manufacture of traditional “*zhourat*” in Beirut. **(A)** boxes with herbal ingredients used in the “*zhourat*” blend; **(B)** cinnamon (*Cinnamomum verum*) is among the spices occasionally used in “*zhourat*” blends; **(C)** *Rosa damascena* flower buds; **(D)** “*zhourat*” bag from the Beirut herbal store showing the role of flowers as a source of color. Photos: **(A)**, **(B)**, **(D)**, L. Attieh; **(C)**, D. Rivera.

The thirty seven samples from Greece (22 traditional mixtures and single ingredients and 15 packed industrial samples), were acquired in different traditional herb stores of Greece: Rethymno and Heraklion in Crete, Naxos, Santorini, Thessaloniki, and *Varvakeion* market in Athens between 2016 and 2019 (Figure 1). The six traditional samples from Syria, were acquired in herbalist local stores of Damascus in 1999 and the four industrial, throughout online commerce since 2011 (Figure 1). In Lebanon, “*zhoura*t”, a renowned herbal blend was studied, six samples of traditional formulations were analyzed three were prepared by three independent informants in Kfarhamam (in Southern Lebanon). One sample was blended in the “*Dabbous*” specialist shop of herbs and spices, in Tripoli (Northern Lebanon). The other two traditional mixtures, which are prepared in Beirut (Figure 2), were acquired from a Lebanese food store in Paris (Les Délices D'Orient). On-line commerce industrial “*zhourat*” samples include Abido Libanesischer Kräutertee, Abido Mills Herbal Tea and London Tea (Lebanon), Al Rayan Zhourat, Zhourat Shamia, Syrian Natural Products Zhourat tea, and Alattar Zhourat Lebnania (Syria). A traditional mixture made by the Assyrian of Tur-Abdin (Abdalla 2004) was also included. Two traditional mixtures from Iran and four from Turkey were obtained from herbalists at Tehran and Istanbul respectively. Industrial samples from Iran (fifteen) and Turkey (five) were obtained at the World’s Expo Food Fair of Milan (Italy), in 2015 (Figure 1), and from different on-line commerce sites in 2020. The outgroup of spices mixtures include: Arabian Spice mixture, Bahrat, Hawayil (Yemen), Berebere and Zahtar (Ellen’s Kitchen, 2020), Laebriq, Ras el Hanut and Tjalaet (Morocco) (González et al., 2010), Ras el Hanout (Morocco) (Bellakhdar, 1997) and Arba'in (Palestine) (Crowfoot and Baldensperger 1932).

Voucher specimens of the samples and herbal ingredients from Syria and Lebanon were deposited at the MUB herbarium (Spain) (NYBG 2020a), while the rest were deposited in the UMH herbarium (Spain) (NYBG 2020b) (Detailed information is available as Supplementary Table S1).

### Botanical Study of Plant Material

In order to separate the ingredients within the mixtures, a nested column of sieves of decreasing diameter of 2, 1 and 0.37 mm, has been used. After sieving, the different components were separated using stainless steel pointed tweezers and a binocular microscope (Olympus; Model: VMT; Illumination source: TL2). Morphological characters of the ingredients were studied with two binocular microscopes: Olympus, SZ-PT (18x–110x) and Bausch and Lomb, StereoZoom, 4 (7x–30x) that are usually employed in plant taxonomic research and pharmacognosy work. The use of one or the other binocular was selected based on the detail of the characteristics to be analyzed. The former has been of considerable help in separating the smaller components and identifying others. The photography of the samples was carried out systematically. Depending on the date, different cameras were used: Fujifilm XQ2 equipped with Fujinon 4X f = 6.4–25.6 mm lens with a built-in flash, Canon EOS M100 equipped with Canon 15–15 mm lens. With image stabilizer and built-in flash and Nikkon D80 equipped with Micro Nikkor 60 mm 1:28 G ED lens. With Niddin digital MF 18 Macro ring flash.

For the determination of Greek herbal mixtures, we have consulted keys and illustrations of different Greek floras (Fielding et al., 2005; Alibertis, 2007; Dimopoulos et al., 2020) and especially Papanikolau and Kokkini (1982) and Aneva et al. (2019) for *Sideritis* species. We paid special attention to *Sideritis* diversity and included in the study monospecific comparison samples, since *Sideritis* are commercialized in the markets of Greece in form of entire plants in excellent state of conservation (Supplementary Tables S1 and S2). We followed the methodology developed at our laboratory for the study of Section *Sideritis* (Rivera et al., 1990; Obon and Rivera, 1994). Intra-population and inter-population genetic and morphological variability in *Sideritis* species is high, something that is well visible in the species with the largest distribution area (Papaporfyriou et al., 2020; Patelou et al., 2020). Kalivas et al. (2014) proposed a molecular tool for taxonomic identification of *Sideritis*, however, only *S. perfoliata* and *S. athoa* differed from the rest with a high level of support. Nevertheless morphometric data, i.e., length of the acumen in the bracts, trichome types, allow to distinguish the different *Sideritis* taxa of the Balkans and Eastern Mediterranean (Karousou et al., 1992; Sahin et al., 2008; Aneva and Zhelev, 2019).

Numerous references were consulted for identifying samples from Eastern Mediterranean and West Asia (Post and Dinsmore, 1932; Mouterde, 1966; Mouterde, 1970; Mouterde, 1983; Tohme and Tohme, 2007; Université Saint-Joseph de Beyrouth, 2009; Zurayk and Talhouk, 2009). We also consulted Nehmé (1980) and the Encyclopedia of Medicinal Plants (in Arabic) (Hayek, 1996; Hayek, 1997; Hayek, 1998a; Hayek, 1998b). Also we consulted other botanical literature of the zone such as Flora Palaestina (Zohary, 1966a; Zohary, 1966b; Zohary, 1972a; Zohary, 1972b; Feinbrun-Dothan, 1977; Feinbrun-Dothan, 1978; Feinbrun-Dothan, 1986a; Feinbrun-Dothan, 1986b) and Flora of Turkey (Davis, 1965; Davis, 1972; Davis, 1982). The Herbarium and living specimens grown in the gardens of the Murcia University (MUB) were referred to as well.

Some of the species that were identified in the mixture and are usually used globally, were verified following Bisset (1994) and Wichtl (1984). Plant names and authors abbreviations were standardized consulting TPL (2020) as it was recommended by Rivera et al. (2014). GRIN-Taxonomy (Wiersema and Schori 2020) was consulted for cultivated plant species. In addition to information on scientific nomenclature, IPNI (2020), POWO (2020), and GBIF (2020) provided distribution maps and images.

The status as wild, cultivated or imported of the different identified botanical taxa was determined from the local floras and other botanical sources consulted above mentioned.

### Data Analysis

In order to determine how different these 93 samples are, we took the 227 ingredients (201 species) as a reference. This led us to build a simplified presence/absence matrix reduced to 83 herbal tea samples and 10 spices mixtures (as outgroup) totalizing 93 individuals, and 201 species (variables). The crude matrix of presence/absence of species was used to compute a dissimilarity matrix using Darwin 6 V.6.0.9 (2015-04-15) (Perrier et al., 2003; Perrier and Jacquemoud-Collet, 2006). The Sokal-Sneath dissimilarity index was calculated (un2) (Eq. 1).

$$d_{ij}=\;\frac{2\left(b+c\right)}{a+2\left(b+c\right)}$$ (1)

Where d_ij_ is the dissimilarity between samples i and j, a: number of variables where x_i_ = presence and x_j_ = presence, b: number of variables where x_i_ = presence and x_j_ = absence and c: number of variables where x_i_ = absence and x_j_ = absence. Dissimilarities are even and are Euclidean distances. The dissimilarity is = 0 for two samples sharing the 201 species and = 1 for two samples which present 0 species shared. This index concerns “presence/absence” data where only “presence” modality is informative, modality “absence” expressing mainly an absence of information. These two modalities are not symmetrical and their exchange leads to a completely different dissimilarity value. This index considers that a common absence for two units is uninformative to measure their dissimilarity (Perrier and Jacquemoud-Collet, 2006). Therefore, similarity here reflects the number of coinciding species and dissimilarity is inversely proportional to this.

These pairwise dissimilarities can be represented in a multidimensional space, but, in order to obtain meaningful graphic representation of these relationships in a two-dimensional plane, we used cluster analysis.

Cluster analysis is a term used to name a set of numerical techniques in which the main purpose is to divide the objects of study into discrete groups. These groups are based on the characteristics of the objects (Kovach, 2007). We used the agglomerative hierarchical method that arrange the clusters into a hierarchy so that the relationships between different groups are apparent. Minimum variance clustering (Ward’s method) focuses on determining how much variation is within each cluster. In this way, the clusters will tend to be as distinct as possible, since the criterion for clustering is to have the least amount of variation (Kovach, 2007). Ward’s method produces a single tree.

To determine the existence of association between species based on the coincidence of the species in samples, we proceeded to transpose the previous matrix of samples x species into one of species per sample. This led us to a transformed presence/absence matrix with 201 individuals (species) and 93 variables (83 herbal samples and 10 spices mixtures).

## Results

### Relevant Species and Plant Families

The study has shown a total of 227 floral and non-floral ingredients belonging to 201 species that were used to prepare the analyzed herbal teas in Greece and the Eastern Mediterranean (Table 1). This difference is due to the fact that several species contribute more than one ingredient used together or separately: *Origanum dictamnus* flowers and leaves; *Citrus* and *Rosa* spp., flowers and fruits, for example. The maximum number of ingredients in one sample was found in the “*arbain*” spice mixture from Palestine and in the “*sarantha*” herbal formula acquired in Naxos (Table 1). Overall samples from Turkey and Iran were shown as less complex. The mean number of ingredients per sample is near 10 (Table 1), being especially rich in ingredients the “*zhourat*” that approaches the levels of Arabian style spices mixtures.

**TABLE 1:** Types and frequencies of plant-based ingredients.

	Zhourat	Arab spices	Greece	Turkey	Iran	Whole set
Samples	19	10	37	10	17	93
Ingredients	76	78	93	42	38	227^a^
Maximum number of ingredients per sample	33	40	40	11	17	40
Average number of ingredients	11	16	12	8	6	10.5
% Samples traditional/total samples	65	50	60	44	12	48
Average number of ingredients traditional	14	24.8	28	9.5	14	17.5
Average number of ingredients industrial	6.3	7.4	7.7	7	4.8	6.5
Food	39	67	44	32	29	127
Non-food	36	11	49	10	9	96
Cultivated	14	30	29	14	19	64
Cultivated and imported	0	1	0	1	1	2
Feral and cultivated	4	2	0	0	1	5
Imported	12	17	13	15	8	30
Wild	26	10	22	0	2	59
Wild and cultivated	20	18	29	8	7	63

^a^ Four ingredients were synthetic chemical substances.

Most ingredients are from plants (223) while chemicals were only four (tricalcium phosphate, citric acid, 3,5-dimethyl-1,2-cyclopentanedione and l-ascorbic acid) in one single Turkish industrial sample. The ingredients consumed as food, whether as a nutrient, condiment or spice, add up to 127, which is 56% of the total. Imported ingredients are only a 13.2% of the total (Table 1), while those from wild or cultivated species account in higher and similar proportions, c. 26–28% (Table 1).

The industrial, online commercialized herbal tea mixtures independently of the style or region from where they come show a significantly lower number of ingredients (Table 1).

Flowers are the more frequently used as a plant ingredient (Table 2) especially in the “*zhourat*” mixtures of Lebanon and Syria and in the Greek herbal mixtures. Fruits surpass flowers in herbal formulations of Iranian herbal teas and, especially in spice mixtures where flowers are rare (Table 2). The category of aerial parts includes flowers and flower bracts in case of Lamiaceae such as *Origanum dictamnus* and others.

**TABLE 2:** Parts of the plant from which the plant ingredients come.

Parts	Zhourat	Arab spices	Greece	Turkey	Iran	Total
Flower	24	5	36	12	11	60
Fruit	17	46	23	9	13	75
Leaf	16	5	13	5	8	25
Erial part	15	15	13	3	3	38
Root or rhizome	1	6	4	6	2	9

The Malvaceae, Asteraceae and Lamiaceae provide the largest number of species whose flowers are used in herbal teas. Fabaceae flowers are characteristic of “*zhourat*” (Table 3). However when we focus on the most relevant species, the number of samples/species index (Table 3) presents maximum values for the Myrtaceae, Rosaceae and Boraginaceae.

**TABLE 3:** Number of species of the plant families that provide floral ingredients.

	Geographical origin							
Families	Zhourat	Arab spices	Greece	Turkey	Iran	Species	Samples	Samples/species
Malvaceae Juss.	5	0	6	4	4	**13**	58	4.5
Asteraceae Bercht. and J.Presl	2	0	**11**	1	1	12	46	3.8
Lamiaceae Martinov	3	1	**10**	1	1	12	53	4.4
Fabaceae Lindl.	**6**	0	1	0	0	6	14	2.3
Rosaceae Juss.	1	2	2	**3**	2	4	37	9.3
Oleaceae Hoffmanns. and link	1	0	1	1	0	2	3	1.5
Poaceae Barnhart	2	0	0	0	0	2	12	6
Rutaceae Juss.	1	0	1	0	1	2	9	4.5
Adoxaceae E.Mey.	1	0	1	0	0	1	6	6
Boraginaceae Juss.	0	0	0	0	1	1	7	7
Caryophyllaceae Juss.	0	0	1	0	0	1	1	1
Iridaceae Juss.	0	1	0	0	1	1	6	6
Lythraceae J.St.-Hil.	0	0	0	1	0	1	2	2
Myrtaceae Juss.	1	1	1	1	0	1	10	10
Plantaginaceae Juss.	1	0	0	0	0	1	1	1

Bold character mark relevant numbers.

Damascus rose and chamomile are the species whose flowers characterize the herbal tea mixtures of Greece and the eastern Mediterranean (Table 4).

**TABLE 4:** Plant species that provide floral ingredients and number of samples in which they appear.

Species	Family	Total samples	Zhourat	Arab spices	Greece	Turkey	Iran
*Rosa* × *damascena* Mill.	Rosaceae Juss.	32	19	1	2	3	7
*Matricaria chamomilla* L.	Asteraceae Bercht. and J.Presl	28	15	0	8	2	3
*Zea mays* L.	Poaceae Barnhart	11	**11**	0	0	0	0
*Alcea damascena* (Mout.) Mout.	Malvaceae Juss.	10	**10**	0	0	0	0
*Alcea setosa* (Boiss.) Alef.	Malvaceae Juss.	7	**7**	0	0	0	0
*Spartium junceum* L.	Fabaceae Lindl.	5	**5**	0	0	0	0
*Cercis siliquastrum* L. ssp*. siliquastrum*	Fabaceae Lindl.	4	**4**	0	0	0	0
*Citrus medica* L.	Rutaceae Juss.	3	**3**	0	0	0	0
*Sambucus nigra* L.	Adoxaceae E.Mey.	6	4	0	2	0	0
*Lavandula angustifolia* Mill.	Lamiaceae Martinov	11	3	1	3	0	4
*Mentha pulegium* L.	Lamiaceae Martinov	5	0	2	3	0	0
*Syzygium aromaticum* (L.) Merr. and L.M.Perry	Myrtaceae Juss.	10	1	6	2	1	0
*Crocus sativus* L.	Iridaceae Juss.	6	0	3	0	0	3
*Echium amoenum* Fisch. and C.A.Mey.	Boraginaceae Juss.	7	0	0	0	0	**7**
*Hibiscus sabdariffa* L.	Malvaceae Juss.	17	0	0	6	7	4
*Tilia cordata* Mill.	Malvaceae Juss.	8	0	0	2	3	3
*Origanum dictamnus* L.	Lamiaceae Martinov	10	0	0	**10**	0	0
*Sideritis raeseri* Boiss. and Heldr.	Lamiaceae Martinov	9	0	0	**9**	0	0
*Sideritis syriaca* L.	Lamiaceae Martinov	9	0	0	**9**	0	0
*Citrus aurantium* L.	Rutaceae Juss.	6	0	0	3	0	3
*Sideritis euboea* Heldr.	Lamiaceae Martinov	7	0	0	**7**	0	0
*Sideritis scardica* Griseb.	Lamiaceae Martinov	6	0	0	**6**	0	0
*Malva sylvestris* L.	Malvaceae Juss.	5	0	0	3	2	0
*Thymbra capitata* (L.) Cav.	Malvaceae Juss.	3	0	0	2	1	0
*Calendula officinalis* L.	Asteraceae Bercht. and J.Presl	4	0	0	**4**	0	0
*Achillea ligustica* Vis. ex Nyman	Asteraceae Bercht. and J.Presl	3	0	0	**3**	0	0
*Sideritis clandestina* (Bory and Chaub.) Hayek	Lamiaceae Martinov	3	0	0	**3**	0	0

Bold character mark relevant numbers. Species included are only those found in three or more samples.

### Main Uses of the Mixtures

**FIGURE 3:**
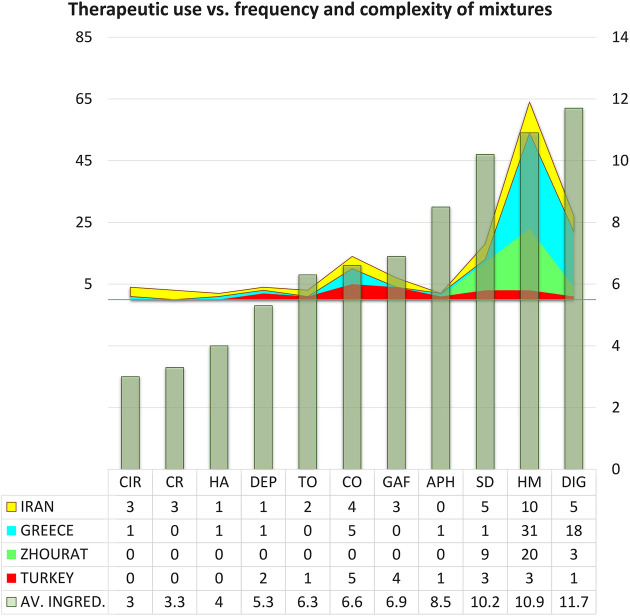
Main categories of traditional uses of the analyzed herbal mixtures. **Axes: X** = Categories of therapeutic use. Abbreviations: CIR, circulatory; CR, anticancer; HA, headache; DEP, depurative; TO, tonic; CO, treatment of common cold; GAF, has a good aroma and flavor; APH, aphrodisiac; SD, sedative; HM, good for health, herbal teas, consumed to preserve health and prevent diseases; DIG, digestive, good for stomach. **Y** (right, 0–12) **Foreground:** Complexity of mixtures used for each specific category. Abbreviations: Data: AV. INGRED, average number of ingredients. **Y** (left, 0–65) **Background**: Number of samples analyzed from each country which belong to each category of use.

In general, most blends are intended for non-specific use as “good for health” or as a digestive after meals. Complexity of mixtures is highest (average number of ingredients above ten) in those used as digestive, good for health (consumed to preserve health and prevent diseases) or as sedative. It is also to these pathologies that a greater number of the analyzed mixtures are destined (Figure 3).

Common cold follows in number of samples but with an intermediate complexity, near seven ingredients on average. Complexity present a minimum (average number of ingredients below or equal to four) in those used to treat circulatory system complications or headache, or to prevent cancer.

The comparison of the proportion of medicinal uses reported by our informants for traditional herbal tea mixtures and those reported in the labels and advertising of simplified industrial versions offer interesting results.

Herbal tea mixtures consumed to preserve health and prevent diseases, or to treat circulatory troubles, or as a sedative, digestive, depurative, aphrodisiac, or tonic, present similar frequencies in both groups.

However differences in frequencies were registered, with a notable higher proportion among the industrial version, in the case of teas consumed merely for their good aroma and flavor (6:1), to treat common cold (4:1). Anticancer uses and the treatment of headache were only recorded in the advertising of industrial blends.

### Geographical and Cultural Patterns

#### Species Characteristic of Greek, Turkish, Lebanese, Syrian and Iranian Styles

**FIGURE 4:**
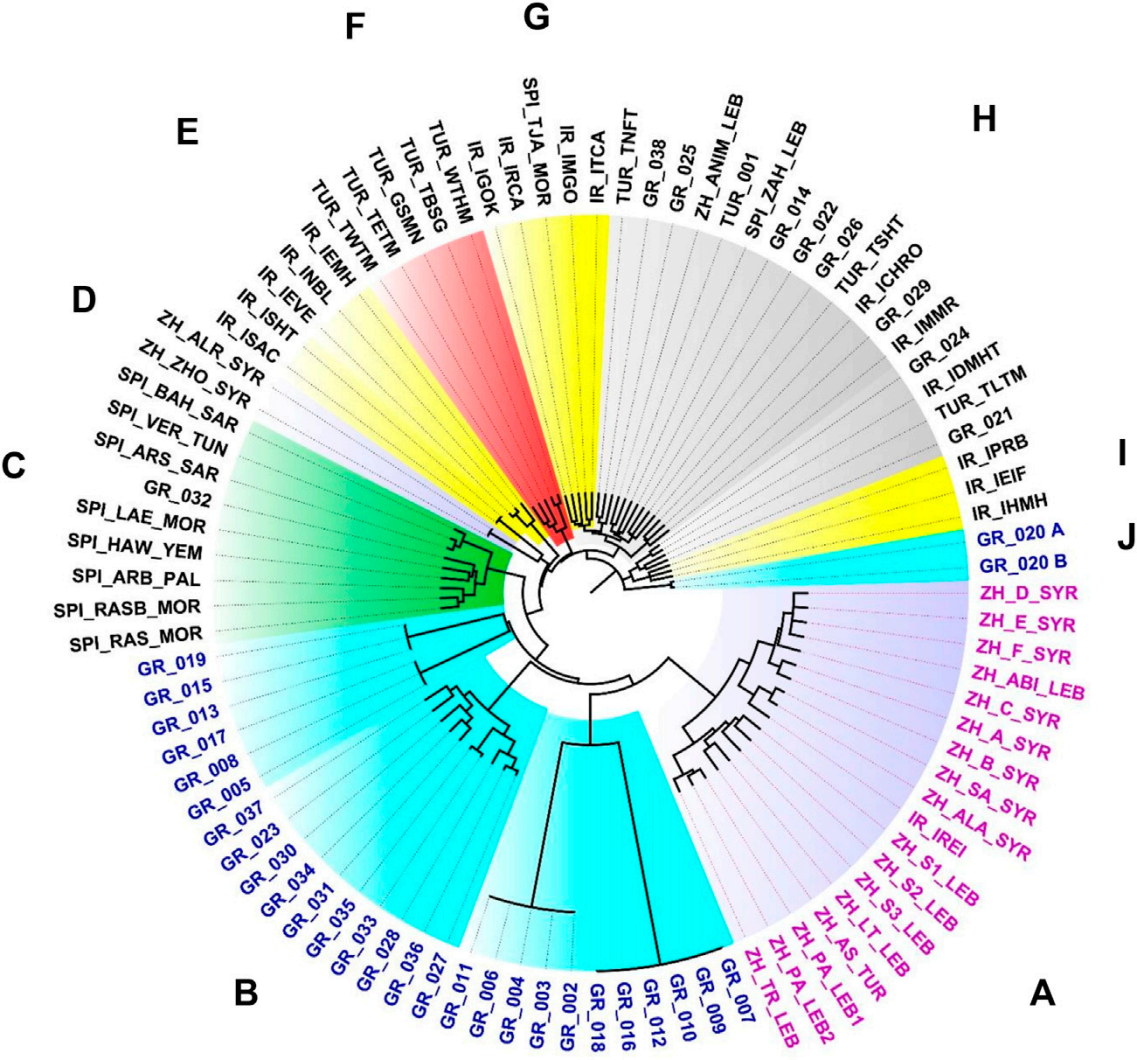
Ward’s minimum variance tree with geographical clusters of herbal tea samples. A, “*Zhourat*” from Lebanon and Syria; B, Main group of Greek herbal mixtures; C, Samples of Arabian style spice mixtures; D, Abnormally poor in ingredients “*zhourat*” commercial samples; E, Iranian samples extremely poor in ingredients, but with *Echium amoenum* Fisch. and C.A.Mey., flowers; F, Turkish samples with cinnamon bark, *Hibiscus sabdariffa*, and ginger; G, Iranian samples with, among others, *Thymus vulgaris* L. and *Lavandula angustifolia* Mill.; H, samples of different origins with no regional differential markers; I, Iranian samples with, among others, *Citrus aurantiifolia* (Christm.) Swingle fruits; J, Complex “*saranda*” mixture from Naxos, with 40 ingredients. Abbreviations for sample codes: GR_, Greek samples; IR_, Iranian; SPI_, spices outgroup; TUR_, Turkish; ZH_, Lebanese and Syrian “*zhourat*”. Image: D. Rivera.

As indicated above, roses and chamomile flowers, both cultivated, are common in herbal teas throughout the territory studied. The differential presence of other species determines peculiar patterns in the composition of the infusions of the different countries, which allow identifying differentiated local styles. Using the ensemble of 201 species the clusters obtained (Figure 4) follow a distinct geographical pattern for over 75% of analyzed samples. Around a 25% of samples for the different areas overlap showing a poorly defined style (Figure 4, cluster H).

The “*zhourat*” is a characteristic flower tea from Lebanon and Syria (Figure 4, cluster A), which in addition to chamomile and rose flower buds, contains corn stigmas, flowers of different species of *Alcea* and as a singular fact the presence of flowers of various species of Fabaceae [*Astragalus* sp., *Cercis siliquastrum* L. subsp. *hebecarpa* (Bornm.) Yalt., and subsp. *siliquastrum, Colutea cilicica* Boiss. et Bal., *Cytisopsis pseudocytisus* (Boiss.) Fertig., and *Spartium junceum* L.]. We have not found any Fabaceae flowers in the Iranian and Turkish samples. In only one sample from Greece we have identified the use of Fabaceae flowers, in this case the inflorescences of *Ebenus sibthorpii* DC. Two abnormally poor in ingredients “*zhourat*” samples cluster together apart from the rest (Figure 4, cluster D).

The Arab spice mixtures act here as outgroup (Figure 4, cluster C), which typically presents cloves and saffron as characteristic floral ingredients (Table 4).

Analyzed Greek herbal teas (Figure 4, clusters B and J) present the higher diversity of ingredients and notably flowers (Table 2). Lamiaceae and Asteraceae predominate among the species that make up Greek herbal teas (Table 3). *Origanum dictamnus* and different *Sideritis* species characterize mixtures from Greece (Table 4), in particular from Crete Island. Lamiaceae flowers are often used within the set of aerial parts in combination with leaves and stems. In many cases the part that is used in the Lamiaceae is mainly, or exclusively, the leaf (rosemary, *Rosmarinus officinalis* L., sage *Salvia fruticosa* or *S. officinalis* L., peppermint, *Mentha* x *piperita* L., or lemon balm, *Melissa officinalis* L., for example).

Turkish teas (Figure 4, cluster F), some within the Ottoman tradition or “*osmnali*” style, often contain *Hibiscus sabdariffa* and jasmine flowers, cinnamon bark and ginger rhizome.

**FIGURE 5:**
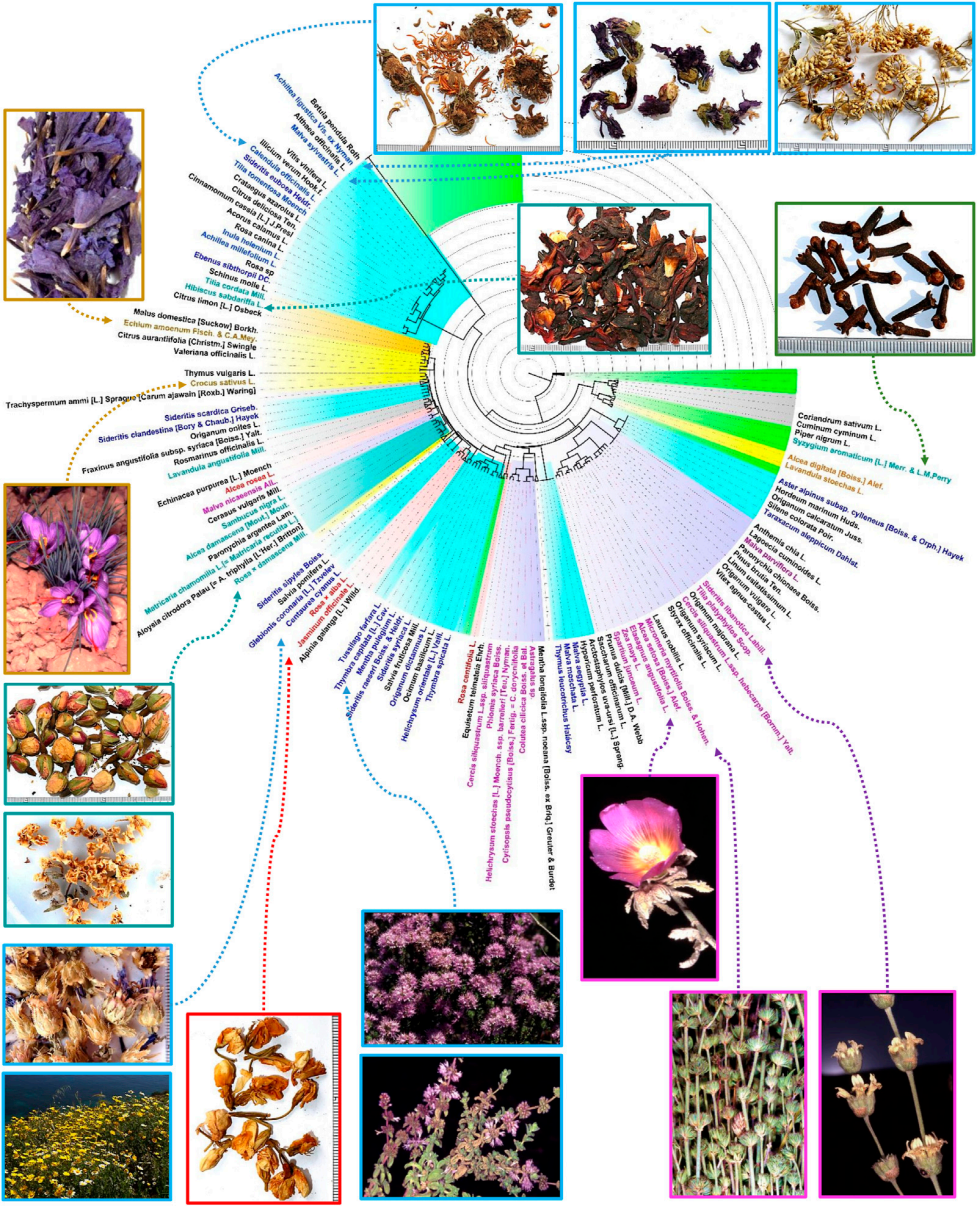
Ward’s minimum variance tree with clusters displaying the association between species. Note: Branches fully composed of non-floral ingredients appear collapsed in the tree. Color codes based in the correlation of species with their geographical provenance: Blue, Greek samples; Magenta, “*Zhourat*”; Red, Turkish samples; Yellow and golden, Iranian samples; Green, Arabian style spices mixtures; Olive, species appearing in the different geographical groups. Black, species furnishing non floral ingredients. Images: C. Obón and D. Rivera.

Saffron stigmas are present in Iranian herbal teas (Table 4; Figure 5), this is not surprising considering that Iran largely produces the world’s annual saffron crop, more than 90% of it, reaching 430 tons in 2019 (Statista, 2020). Among the flowers used in Iranian mixtures, those of *Echium amoenum* stand out for their uniqueness and high frequency of use (Table 4; Figure 5). Several extremely poor in ingredients Iranian samples cluster together in reason of the presence in these of *Echium amoenum* (Figure 2, cluster E). Iranian samples with, among others, *Thymus vulgaris* L. and *Lavandula angustifolia* Mill. conform cluster G (Figure 4). Cluster I (Figure 4) includes three Iranian samples characterized by the presence of *Citrus aurantifolia* (Christm.) Swingle roasted fruits, more or less fragmented, with dried apple and *Rosa* × *damascena* flowers.

#### Association of Species Within the Herbal Teas

The most characteristic association of species among the floral ingredients and that occur in the different styles analyzed, especially in the “*zhourat*” is that of the Damascus rose (*Rosa* × *damascena*) with chamomile (*Chamomilla matricaria*) (Table 4). *Aloysia citrodora* Palau [ = *A. triphylla* [L’Her.] Britton] leaves often appear together with the above (Figure 5).

Given the heterogeneity in the number of samples analyzed within each cultural context and their ingredients it is difficult to detect associations between species, especially when we focus exclusively on flowers. If we include all types of ingredients then several associations appear (Figure 5):

1. *Rosmarinus officinalis* aerial parts/*Lavandula angustifolia* flowers
2. *Astragalus* sp./*Colutea cilicica* Boiss. et Bal./*Cytisopsis pseudocytisus* [Boiss.] Fertig. in “zhourat” samples
3. *Origanum dictamnus* L./*Salvia fruticosa* Mill. leaves/*Sideritis syriaca* L., flowers and leaves in Greek samples from Crete.
4. *Alcea digitata* [Boiss.] Alef./*Lavandula stoechas* L. flowers in Iranian samples.

## Discussion

### Diversity of the Formulations

The existence of complex formulations of herbal teas with a high number of ingredients is not unique to the Eastern Mediterranean Region. On the island of Madeira (Portugal), located in the Atlantic, a formula “for all ills” with forty ingredients was listed, with numerous endemic plant species and a parasitic fungus [*Laurobasidium lauri* (Geyl.) Jülich] (Rivera and Obón 1995a; Rivera and Obón, 1995b).

In Traditional Chinese Medicine (TCM), and Kampo Japanese Medicine, there is a large number of classic formulae (typically 10–15 herbal ingredients). It is argued in favor of this complexity that TCM herb formulae are prescribed in such a way that each herb is used to its greatest advantage, which improves the results of the treatment and reduces any adverse effects of the other herbs (Yu et al., 2006). The rationale behind these mixed herbal formula is not merely the quantitative addition of different herbs but the more complex interactions between herbs with different therapeutic functions. There are different possible interrelationships between herbal drugs in a formula such as synergistic, additive and antagonistic (reducing curative effects or detoxifying, or even increasing toxicity) (Guimarães et al., 2011). There are also instances in TCM of the use of “unusual” formulas containing peculiar herbal ingredients or a large number of herbs, i.e., more than 50 ingredients, for which no rationale is available (Jia et al., 2004).

In the set of samples analyzed, the more traditional and less industrial ones present a markedly greater number of ingredients (Table 1) that are slightly minced, therefore whole or in coarse fragments instead of crushed in powder, as usual in industrial tea bags. This implicates a random formulation of each single dose, where only predominant ingredients will be present with probability near 1 while others would rarely be present. This phenomenon was verified even in larger samples. From the “*sarantha*” formula bough in Naxos, we acquired two independent subsamples of c. 200 g in two consecutive days, both from the same bag of c. 5 kg, one was found to have 40 ingredients while the other only contained 38. This extreme diversity of single doses has a biological meaning that requires further investigation since simply attributing it to ignorance is not a sufficient and satisfactory explanation.

What stands as primary evidence from this study is that industrial processing and commercial versions tend to drastically reduce the diversity of traditional herbal tea formulations producing simplified versions. The reduction ratio in some cases is dramatic, going from thirty ingredients to three, in the case of the Lebanese and Syrian “*zhourat*”, or from forty to twelve of fewer ingredients, in the case of the Greek “*sarantha*”.

In parallel with the above, the use of local wild and feral endemic species is reduced to nearly zero in industrial herbal teas that depend almost exclusively on cultivated or imported materials. However, the examples of *Alcea* spp. in Lebanon (Nisrine at al., 2016) and *Echium amoenum* in Iran (Latmahalleh et al., 2011) show that cultivation of these formerly wild species is possible, allowing their wider use in commercial preparations.

The cultivation of the most used herbs enable their standardization, which is crucial for such herbal products, and reduces the impact of gatherings on natural populations of the species that often are overexploited. And, last but not least, if done in the local communities would contribute to the incomes of the families in the area.

### The Medicinal Potential of the Most Characteristic Flowers

#### Adoxaceae

In the present study, analyzed blends (Supplementary Tables S1 and S2) containing *Sambucus nigra* L. flowers are used, according to our informants, to prevent diseases, to treat common cold and as a sedative. However the small proportion of the elderflowers in the analyzed blends, where they are mixed with a dozen other ingredients, made it difficult to get an idea of its contribution to the medicinal effect of the blend.

In the Aegean Islands, flowers and fruits boiled are used for bronchitis, cough, and fever and as expectorants (Axiotis et al., 2018). An elderflowers decoction is used in Central Macedonia (Greece) against inflammation of the respiratory tract, to treat cough or as expectorant [also combined with other ingredients (Tsioutsiou et al., 2019)]. Güzel et al. (2015) mention the use of elderflowers infusion in the treatment of bronchitis and cough in Hatay Province (Turkey) and Latifian and Funda Arslanoğlu (2018) the use in the Azerbaijan Region of Iran for cold and, in general, for respiratory troubles.

The flowers of elderberry contained tenfold more flavonols (214.25 mg/100 g) than fruits (20.18 mg/100 g) and several times more than the leaves (17.01 mg/100 g). Phenolic acids in the elderberry fruits were less diverse than those present in flowers. Phenolic acids and flavonols in teas from elderflowers were relatively stable compounds during their 21-month of storage. Elderflower extract at a concentration of 252 μg/ml inhibited the influenza A virus (H1N1)-induced Madin–Darby canine kidney (MDCK) cell infection (Sidor and Gramza-Michałowska, 2015). Infusions of elderflowers are a good traditional remedy for many kinds of diseases, such as, for the treatment of inflammation, joint pain, skin disorders, colds, fever, respiratory disturbances, asthma treatment and as a diuretic (Salvador et al., 2019; Ferreira et al., 2020). Elderflower’s traditional use for the relief of early symptoms of common cold has been found to fulfill the requirement of medicinal use for at least 30 years according to the Committee on Herbal Medicinal Products of the European Medicines Agency (Salvador et al., 2019).

#### Asteraceae

*Matricaria chamomilla* L (= *M. recutita* L.) is one of the most commonly consumed herbal teas worldwide. In the present study (Supplementary Tables S1 and S2), analyzed blends containing *Matricaria chamomilla* flowers are used to prevent diseases, to treat common cold, or headache, and as a digestive, or sedative, or merely consumed as a pleasant tea. In Iranian folk medicine the flowers are taken as teas as antitussive, to treat common cold, colic and menstrual pain (Amiri and Joharchi, 2013). Delfan et al. (2014) report the use in Lorestan (Iran) of chamomile herbal teas for the treatment of migraine. In the Aegean the reported uses of chamomile tea include: anti-inflammatory, sedative, antispasmodic and vasodilator (Axiotis et al., 2018). In Thessalonica (Greece) the uses were numerous, antiemetic, bloating, diarrhea, dyspepsia, gall disorders, jaundice, spasmolytic, allergy, influenza, obesity, sedative, eye inflammation, common cold, arthritis, rheumatisms, antiseptic, eczema, hair tonic, infections of the vagina, menstruation disorders, antipyretic and appetizer (Hanlidou et al., 2004). These coincide with the general uses recorded by Singh et al. (2011) and those mentioned from Mardin (Turkey) by Akgul et al. (2018). Akgül et al. (2016) mention the use in Cappadocia (Turkey) of chamomile teas as a sedative, analgesic and to treat sinusitis.

The most important bioactive components are the flavonoids and essential oil constituents (Tschiggerl and Bucar, 2012). Sotiropoulou et al. (2020) have studied by LC-MS aqueous chamomille extracts. The major phenolic compounds were rutin trihydrate, ferulic acid, chlorogenic acid, and apigenin-7-O-glucoside. The optimum extraction temperature was at 80 C with maximal antioxidant activity and the highest total phenolic content, which is coherent with its traditional use in teas.

#### Boraginaceae

In the present study, analyzed blends containing *Echium amoenum* Fisch. and C.A.Mey. flowers, are used to prevent diseases, and as a sedative, digestive, circulatory, tonic and to treat common cold. *Echium amoenum* is called “*Gav Zaban*” in Persian and “*Lesan-al-sour*” in Iranian traditional medicine. The flowers are used in single-species teas in Iranian traditional medicine and have been widely used for a variety of purposes, such as sedative, anxiolytic, demulcent, anti-inflammatory, antihypertensive, analgesic, tonic, anti-infective, febrifuge, and antioxidant. It was also mentioned for the treatment of nephrolithiasis and headache (Amiri and Joharchi, 2013; Rouhi-Boroujeni et al., 2016; Azizi et al., 2018).

Pharmacological and preliminary clinical investigation on *Echium amoenum* flower extracts focusing on nervous and degenerative disorders offer promising evidence for: obsessive-compulsive disorder (Sayyah et al., 2009), antidepressant effects (Sarris et al., 2011), Parkinsonism (Sadeghi et al., 2018a), Alzheimer’s (Sadeghi et al., 2018b), and anxiolytic (Rabbani et al., 2004; Rabbani et al., 2011). However it was experimentally evidenced that *Echium amoenum* causes hepatotoxicity and nephrotoxicity in dose and time dependent effects (Ghorbani et al., 2020). This advises against its prolonged consumption in monospecific teas, however its presence in mixtures would reduce the risk due to the decrease in the dose. A relatively low temperature of the water would improve aroma and other organoleptic properties of *Echium amoenum* flower tea (Amanpour et al., 2019).

#### Fabaceae

In the present study, analyzed blends containing Fabaceae flowers [*Cercis siliquastrum* L., *Colutea cilicica* Boiss. et Bal. *Cytisopsis pseudocytisus* (Boiss.) Fertig., *Ebenus sibthorpii* DC. or *Spartium junceum* L.] are used as a digestive, sedative, and to prevent diseases.

Numerous are the Fabaceae species used in traditional medicine of Iran, however flowers are not used (Amiri and Joharchi, 2013; Delfan et al., 2014), a similar situation occurs in the Balkans and other countries of West Asia. However, the flower tea of *Ononis talaverae* Devesa and G. López is used in the Mount Hermon area (Lebanon) to treat urinary disorders and kidney stone (Baydoun et al., 2015). Tsioutsiou et al. (2019) report the use in Central Macedonia (Greece) of a decoction of *Cercis siliquastrum* flowers against joint pains and rheumatisms.

*Spartium junceum* L. bark, shoots and seeds are used in traditional medicine of the Egean but the use of flowers is not recorded (Axiotis et al., 2018). *Spartium junceum* L. Menghini et al. (2006) results reveal that flowers extract show marked analgesic activity and the absence of gastric ulcerogenic activity. The phytochemical investigation detected the presence of flavonoids (flavones and isoflavones), saponins, and quinolizidine alkaloids. The hydroalcoholic extract of *S. junceum* flowers strongly inhibited B16-F10 murine melanoma cell proliferation, while just a feeble effect was observed on C2C12 murine myoblasts (Nanni et al., 2018).

The occasional presence of *S. junceum* flowers in “*zhourat*” teas could be due to their analgesic activity.

#### Iridaceae

In the present study, analyzed blends containing *Crocus sativus* L. stigmas are used to prevent diseases, or as a sedative, and to treat common cold and headache. The stigmas, saffron, are traditionally used in Iran as tonic, emmenagogue, aphrodisiac and digestive (Amiri and Joharchi, 2013). In Thessalonica their uses include dyspepsia, liver disorders, anti-cancer, sedative, teething pains, bronchitis, pneumonia, dysmenorrhea, aphrodisiac, and stimulant (Hanlidou et al., 2004). Their main use is as a condiment that impart yellow color and a peculiar flavor and aroma to traditional dishes not only in Iran, the major saffron producer, but along whole West Asia and the Mediterranean. Akgül et al. (2016) mention the use of saffron flowers (without specifying whether these were whole or only styles) tea in Cappadocia (Turkey) as appetite stimulant, digestive and diuretic.

Avicenna described various uses of saffron, including its use as an antidepressant, hypnotic, anti-inflammatory, hepatoprotective, bronchodilator, aphrodisiac, inducer of labor, emmenagogue and others (Hosseinzadeh and Nassiri-Asl, 2013).

Saffron contains more than 150 volatile aroma-yielding compounds mainly terpenes, terpene alcohol, and their esters, notably picrocrocin and safranal. *Crocus sativus* styles showed a number of pharmacological activities such as antihypertensive, anticonvulsant, antitussive, anxiolytic, aphrodisiac, antioxidant, antidepressant, anti-inflammatory, sedative. It also improves memory and learning skills, and increases blood flow in retina and choroid (Srivastava et al., 2010). Saffron extract showed effectiveness as antidepressant in clinical trials in comparison to placebo and antidepressant drugs. It was more effective than placebo, and as effective as donepezil in clinical trials on anti-Alzheimer effect. Clinical trials on antipruritic in skin have shown that saffron was more efficient than placebo. Clinical trials conducted on women with premenstrual syndrome showed that saffron could reduce symptoms more than the placebo and similar to standard treatments (Moshiri et al., 2015).

#### Lamiaceae

In the present study, analyzed blends containing *Lavandula angustifolia* Mill. flowers are used to prevent diseases, and as a digestive, sedative, depurative, circulatory tonic, or to treat common cold. Flowers and other aerial parts of this plant are used in traditional medicine of Iran in the treatment of common cold, insomnia and nervous and vascular disorders, however this use is often base on imported material not locally grown (Amiri and Joharchi, 2013; Kırmızıbekmez et al., 2009. Rouhi-Boroujeni et al. (2016) mention the use in the treatment of headache in Iran.

The essential oils from fresh and dried flowers and erial parts of lavender were analyzed by Smigielski et al. (2018). Their main volatile components were linalool (26.5–34.7%), linalyl acetate (19.7–23.4%), β-ocimene (2.9–10.7%), and α-terpineol (2.8–5.1%). The lavender essential oils showed high activity against bacteria (*B. subtilis*, *S. aureus*, *E. coli, P. aeruginosa*), yeast and filamentous fungi (*Candida* sp., *A. niger*, *P. expansum*), inhibiting their growth at concentrations ranging from 0.4 to 4.5 μg/ml. The highest antioxidant activity was exhibited by the essential oil from fresh erial parts (IC_50_ = 77.11 mg/ml) while the oil from dried flower displayed the weakest activity (IC_50_ = 22.1 mg/ml). However the use of lavender flowers in herbal teas is mainly based on dried flowers a weak antioxidant activity due to the essential oil should be expected.

In the present study, analyzed blends containing *Origanum dictamnus* L. flowers are used to prevent diseases, to treat common cold, or headache, and as a digestive or aphrodisiac.

The aerial parts of the plants are used in various preparations for stomach and gastric disorders, as well as for the maintenance of good health. In Thessalonica their tea is used to treat diabetes, liver disorders, spasms, stomach ulcer, high cholesterol levels, headache and dysmenorrhea, and as an antiseptic, diuretic, antibacterial, aphrodisiac, and tonic (Hanlidou et al., 2004). Tsioutsiou et al. (2019) report the use in Central Macedonia (Greece) of a decoction against cough and symptoms of common cold.

Polyphenolic components, flavonoids and coumarins have been identified from the methanol extract of aerial parts of the plant, while from the water extract: coumaric acid, ferulic acid and catechin have been identified too. Furthermore, from the polar extracts of the erial parts of *O. dictamnus*, monoterpenes, alicyclic derivatives, flavonoids and depsides (salvianolic acid P, rosmarinic acid and rosmarinic acid methyl ester) have been isolated and structurally determined (Varsani et al., 2017).

Teas made with 20–30 g of plant material in 0.5–1 L hot water are used as a tonic, anti-convulsion, against tonsillitis, cold, cough, sore throats, diuretic, digestive, spasmolytic, against stomach and kidney discomforts. It has been also recommended traditionally, against liver diseases, diabetes and obesity (Liolios et al., 2010). The overall contribution of *O. dictamnus* flowers is quite small since the most visible element from the inflorescences are the bracts that usually appear together with the leaves in teas.

In the present study, analyzed blends containing flowers (also bracts and leaves) of *Sideritis* spp [*S. clandestina* (Bory & Chaub.) Hayek, *S. euboea* Heldr., *S. libanotica* Labill., *S. raeseri* Boiss. and Heldr., *S. scardica* Griseb., *S. sipylea* Boiss., or *S. syriaca* L.] are used to prevent diseases, and as a digestive, depurative, circulatory, or aphrodisiac, and to treat common cold and headache.

*Sideritis* species have been used in traditional medicine of the Balkans and Eastern Mediterranean for their antimicrobial, antiulcerogenic, digestive and anti-inflammatory properties. In Thessalonica their teas are used to treat dyspepsia, anemia, fever, influenza, and common cold, and as a diuretic, and tonic (Hanlidou et al., 2004). In Mugla (Turkey) *Sideritis* spp. flowers and bracts are used to treat hoarseness and common cold (Gürdal and Kültür, 2013). Tsioutsiou et al. (2019) report the use in Central Macedonia (Greece) of a decoction of *Sideritis scardica* flowers and bracts against inflammation of the respiratory tract and cough. A *Sideritis libanotica* decoction is drunk in Hatay Province (Turkey) as an appetizer, carminative and sedative (Güzel et al., 2015).

*Sideritis* extracts contain essential oils, terpenoids, aliphatic and phenolic compounds and flavonoids (Aneva et al., 2019). The species most often used are: *S. raeseri* (Romanucci et al., 2017); *S. scardica*, which is used in bronchitis and bronchial asthma, common cold and lung emphysema, in the treatment of inflammation, gastrointestinal disorders, coughs, for the prevention of anemia, and have cytotoxic effects (Tadić, et al., 2012; Todorova and Trendafilova, 2014). *Sideritis scardica* and *S. euboe*a present a potential for improvement of memory in healthy adults as well as in dementia (Hofrichter et al., 2016). *S. clandestina* tea prevents anxiety-related behavior (Vasilopoulou et al., 2013).

Stanoeva et al. (2015) show that the polyphenolic profiles in the different Balkan *Sideritis* species that they analyzed responded to a greater extent to environmental factors and geographical origin than to the taxonomic ascription of the sample.

#### Malvaceae

In the present study, analyzed blends containing flowers of *Alcea* spp. are used to prevent diseases and as sedative, and digestive or to treat common cold.

Several *Alcea* species provide flowers used in Iranian traditional medicine to treat cough, fever, and constipation and as a depurative (Amiri and Joharchi, 2013). Lebanese herbalists sell *Alcea setosa* Boiss. flowers for the treatment of the oral cavity (gums) inflammation, stomach ulcers and cough (Deeb et al., 2013). Güzel and Guzelsemme (2018) recorded the use of *A. setosa* floral tea in Hatay (Turkey) for cough, and Akgul et al. (2018), in Mardin Province (Turkey), for cough and cold. Tsioutsiou et al. (2019) report the use in Central Macedonia (Greece) of *Alcea rosea* L. flowers decoction against cough in combination with flowers of *Sambucus nigra*; and decoction against rheumatisms and musculoskeletal pain in combination with flowers of *Robinia pseudacacia* and *Cercis siliquastrum*. Akgül et al. (2016) mention the use in Cappadocia (Turkey) of *A. rosea* floral teas for cold and cough, and Sargin et al. (2015) for bronchitis in Manisa (Turkey). *A. rosea* flowers are used as medicinal in Iraq (Al-Snafi, 2018).

Hanif et al. (2019) results suggest that *Alcea rosea* extracts cause bronchodilation through dual inhibition of phosphodiesterase enzyme and Ca2+ influx, which substantiate its potential in airways disorders. Flavonoids (Dihydrokaempferol-4′-O-β-d-glucopyranoside, dihydrokaempferol, kaempferol-3-O-[6″-(E-coumaroyl)]-β-d-glucopyranoside, kaempferol-3-O-β-d-glucopyranoside, Apigenin and kaempferol-3-O-α-l-rhamnopyranosyl-(1‴→6″)-β-d-glucopyranoside) were isolated from *Alcea rosea* flowers, some showed potent cytotoxic activity against HepG-2 cell line *in vitro*, significant antioxidant activity or an immune stimulant activity (Abdel-Salam et al., 2018).

The analyzed blends containing *Hibiscus sabdariffa* L. flowers are used to prevent diseases, or as a sedative, digestive, depurative, aphrodisiac, or tonic, and to treat common cold, circulatory troubles or merely as a pleasant tea.

With the name “*chai makkeh*” *H. sabdariffa* flowers are used in the treatment of diabetes, hypertension, cardiovascular diseases and stress in Iranian traditional medicine (Amiri and Joharchi, 2013). In Thessalonica are used to treat diabetes, constipation, dyspepsia, cholesterol, obesity, and a hair tonic, depurative and stimulant (Hanlidou et al., 2004). In Hatay Province (Turkey) the decoction of sepals is used for cold and flu (Güzel et al., 2015).

The most valued and widely used part of *Hibiscus sabdariffa* is the fleshy flower calyx, it is rich in citric acid (3.74% hence the sour taste imparted to the teas) pectin, anthocyanin pigments and vitamins. *In vitro* and *in vivo* studies as well as some clinical trials provide some evidence for antibacterial, anti-oxidant, nephro- and hepato-protective, renal/diuretic effect, effects on lipid metabolism (anti-cholesterol), anti-diabetic and anti-hypertensive effects among others (Da-Costa-Rocha et al., 2014). Phenolic acids (protocatechic acid and others), tricarboxylic acids (hydroxycitric acid), sulfoxides (hibiscus acid) and anthocyanins (delphinidin-3-sambubioside and cyanidin-3-sambubioside) are likely to contribute to the reported effects. *Hibiscus sabdariffa* has an excellent safety and tolerability record (Da-Costa-Rocha et al., 2014). Phenolic compounds were better extracted with hot water that also resulted in a higher antioxidant capacity in the extracts (Lim, 2014).

#### Rosaceae

In the present study, analyzed blends containing *Rosa* × *damascena* Mill. flowers are used as a digestive, sedative, aphrodisiac, or depurative, to prevent diseases, to treat common cold and headache, or merely as a pleasant tea.

*Rosa* × *damascena* flowers, “*Gole Mohammadi*”, are used in Iranian traditional medicine in single-species teas mainly as a sedative or laxative (Amiri and Joharchi, 2013). Lebanese herbalists sell the flowers for the treatment of anemia, depression, hoarseness and constipation (Deeb et al., 2013). *R*. × *damascena* flower and its extracts are extremely relevant in the culture of Greece and the Eastern Mediterranean, as a food in itself, and as a main ingredient of traditional herbal teas (Başer et al., 2013). Güzel et al. (2015) mention the use of diluted rose water as a laxative and digestive in Hatay Province (Turkey) but not of rose petals tea.

The flower contains terpenes, glycosides, flavonoids, and anthocyanins that have beneficial effects on human health (Boskabady et al., 2011). Several investigations confirmed that *Rosa damascena* has antioxidant, anti-inflammatory, antiviral (anti-HIV) and antibacterial activities. It is used for numerous digestive problems such as constipation, in this case as a laxative. *R. damascena* eye drops are useful in ophthalmic disorders. *R.* × *damascena* has potential in the treatment of hepatitis or at least a remarkable hepatoprotective activity. Recent scientific studies have confirmed antilipase, anti-Alzheimer’s, alpha-glucosidase inhibitor, and anti-dysmenorrhea activities. Other respiratory and cardiovascular effects are reported. It is suggested that the liposoluble non-polar constituents of this plant are the main responsible for most of the activities mentioned above. The pharmacological effects on the CNS are hypnotics, antidepressants, analgesics and anticonvulsants as well as in the treatment of dementia (Boskabady et al., 2011; Akram et al., 2020). Nayebi et al. (2017) found promising evidences for the effectiveness and safety of Damask rose in pain relief but highlighting the need for appropriate clinical trials to verify these indications.

#### Rutaceae

In the present study, analyzed blends containing *Citrus aurantiu*m L., flowers are used to prevent diseases, or merely consumed as a pleasant tea.

*Citrus aurantium* flowers, “*Bahar Naranj*”, are used in Iranian traditional medicine in single-species teas mainly as a sedative, anxiolytic, digestive, and antihypertensive (Amiri and Joharchi, 2013). *Citrus aurantium* aroma was significantly more active than placebo in treating anxiety in patients with acute coronary syndrome in a double-blind placebo-controlled trial (Moslemi et al., 2019). *Citrus aurantium* flower extract showed to be effective in terms of reduction in preoperative anxiety before minor operation in a double-blind placebo-controlled trial (Akhlaghi et al., 2011).

#### Blends Versus Single-Species Teas

As we discussed previously, the possible interactions between the active principles of each of the ingredients and with those of the rest of the ingredients are very numerous. The effect in no case will be the mere sum of the effects of the ingredients separately. Furthermore, each dose is almost unique due to the stochastic process that governs the presence/absence and proportion of ingredients of each one. If we assume that of an herbal tea made up of 12 ingredients, only 9 are present on average in each singular dose, the number of possible combinations is 220, but if the ingredients in the dose are reduced to 6, the possible combinations rise to 924. In Herbal teas formulated with 40 ingredients, such as the Greek “*sarantha*”, if only 25 of these are incorporated in each specific dose, the possibilities skyrocket to 40 × 10^9^ possible combinations. We can ask ourselves: what biological implication can this randomness have? We have no detected a standard group of species characterizing these “*sarantha*” therefore it seems that it’s the number of 40 ingredients wat matters in local traditions. One can wonder that the fact of obtaining a random composition when sampling a single dose from a tea mixture of 40 different herbs poses the question whether the preparation of a tea was indeed the form of preparation that such multi-ingredient mixtures were originally intended for. Mixtures such as these might have earlier been prepared for alcoholic extracts (medicinal wines and tinctures) or as a hot bath. We discard the use in form of powder mixtures for oral applications since the inflorescences and other plant parts are always found in the analyzed samples entire, without grinding. This leads to a further question, about the motives or background of the composer of the particular herbal tea mixtures investigated.

Many of these mixtures do not have a specific therapeutic use, they are used to improve the general condition of the person and this seems to be related to the presence of numerous species in each blend, belonging to different families of plants, with very different compositions and effects. The ingredients, according to the informants, enter the mixture for various reasons, sometimes it is for giving better flavor, others for the color (Figures 1, 2) and others as a preventive of diseases.

The answer can range from discovering that it is completely absurd, to suggesting mechanisms aimed at preventing the emergence of pathogen resistance, or habituation of treated patients, or the development of cumulative toxicity. In any case, the study of complex floral infusions opens a field that requires new and more comprehensive pharmacological approaches.

## Conclusion

Flowers play a relevant role in traditional and industrial, herbal teas of Greece and the Eastern Mediterranean. Their differential presence is related with local styles of formulating herbal teas but also with the fact that nearly a 30% are from wild local endemic species. Their presence is not merely for aesthetic reasons because of their colors but also especially for the flavor and aroma they impart to the tea and their medicinal properties. Some are well-known (chamomile, Damask rose). However others are still awaiting for detailed pharmacological studies. But what is more necessary is the study of the activities of the whole aqueous extracts and their variability.

Flowers of some species, particularly of Fabaceae, are exclusively used in mixtures, and their use in monospecific herbal teas is not yet recorded.

The possible interactions between the active principles of each of the ingredients and with those of the rest of ingredients are very numerous. The effect in no case will be the mere sum of the effects of the ingredients separately. Furthermore, each dose is almost unique due to the stochastic process that governs the botanical ingredients present in each one. We can ask ourselves: what biological signification can this randomness have? The answer can range from discovering that it is completely absurd, to suggesting mechanisms aimed at preventing the emergence of pathogen resistance, or habituation of treated patients, or the development of cumulative toxicity. In any case, the study of complex floral infusions opens a field that requires new and more comprehensive pharmacological approaches.

The presence in substantial proportion of key ingredients, especially flowers, should be expected in industrial versions addressed to the global market as long as these aim to keep rooted in the local cultural traditions. However at present industrial versions, especially those in form of single dose bags, tend to be extremely simplified, resulting poor in ingredients and clearly denote a degradation of the traditional formulas.

Mislabeling and replacement of ingredients is a supplementary step in the degradation of traditional herbal mixtures. Thus to avoid confusion scientific names should systematically be used along with the vernacular and English names. It is strongly advised to systematically label using scientific names of ingredients.

We draw attention on the urgent need in exhaustively recording in Greece and the Near East, the formulation and use of traditional herbal mixtures and their numerous local variants.

## Data Availability

The datasets presented in this study can be found in online repositories. The names of the repository/repositories and accession number(s) can be found in the article/Supplementary Material.

## References

[B1] Abdalla M. (2004). Wild growing plants in the cuisine of modern Assyrians in the Eastern Syrian-Turkish borderland. J. Assyrian Acad. Stud. 18 (2), 50–58.

[B2] Abdel-Salam N. A. Ghazy N. M. Sallam S. M. Radwan M. M. Wanas A. S. ElSohly M. A. (2018). Flavonoids of *Alcea rosea* L. and their immune stimulant, antioxidant and cytotoxic activities on hepatocellular carcinoma HepG-2 cell line. Nat. Product. Res. 32 (6), 702–706. 10.1080/14786419.2017.1332602 28580799

[B3] Adler L. S. (2000). The ecological significance of toxic nectar. Oikos 91 (3), 409–420. 10.1034/j.1600-0706.2000.910301.x

[B4] Akgul A. Senol S. G. Yildirim H. Secmen O. Dogan Y. (2018). An ethnobotanical study in Midyat (Turkey), a city on the silk road where cultures meet. J. Ethnobiol. Ethnomed. 14 (1), 1–18. 10.1186/s13002-017-0201-8 29415748 PMC5804065

[B5] Akgül G. Yılmaz N. Celep A. Celep F. Çakılcıoğlu U. (2016). Ethnobotanical purposes of plants sold by herbalists and folk bazaars in the center of Cappadocica (Nevşehir, Turkey). Indian J. Traditional Knowledge 15 (1), 103–108.

[B6] Akhlaghi M. Shabanian G. Rafieian-Kopaei M. Parvin N. Saadat M. Akhlaghi M. (2011). *Citrus aurantium* blossom and preoperative anxiety. Braz. J. Anesthesiology 61 (6), 702–712. 10.1016/S0034-7094(11)70079-4 22063371

[B7] Akram M. Riaz M. Munir N. Akhter N. Zafar S. Jabeen F. (2020). Chemical constituents, experimental and clinical pharmacology of *Rosa damascena*: a literature review. J. Pharm. Pharmacol. 72 (2), 161–174. 10.1111/jphp.13185 31709541

[B8] Al-Snafi A. E. (2018). Traditional uses of Iraqi medicinal plants. IOSR J. Pharm. 8 (8), 32–96.

[B9] Alibertis A. (2007). Plantes médicinales, aromatiques et comestibles de Crète. Héraklion: Mystis.

[B10] Amanpour A. Zannou O. Kelebek H. Selli S. (2019). Elucidation of infusion-induced changes in the key odorants and aroma profile of Iranian endemic borage (*Echium amoenum*) herbal tea. J. Agric. Food Chem. 67 (9), 2607–2616. 10.1021/acs.jafc.9b00531 30758196

[B11] Amiri M. S. Joharchi M. R. (2013). Ethnobotanical investigation of traditional medicinal plants commercialized in the markets of Mashhad, Iran. Avicenna J. Phytomed 3 (3), 254–271. 25050282 PMC4075713

[B12] Aneva I. Zhelev P. Kozuharova E. Danova K. Nabavi S. F. Behzad S. (2019). Genus *Sideritis*, section *Empedoclia* in southeastern Europe and Turkey - studies in ethnopharmacology and recent progress of biological activities. DARU J. Pharm. Sci. 27 (1), 407–421. 10.1007/s40199-019-00261-8 PMC6593022 30927208

[B13] Aneva I. Zhelev P. (2019). Morphometric studies of *Sideritis scardica* Grsb. and *S. syriaca* L. in their natural populations in Bulgaria. Bol Latinoam. Caribe Plant Med. Aromat 18 (1), 71–80. 10.35588/blacpma.19.18.1.06

[B14] Axiotis E. Halabalaki M. Skaltsounis L. A. (2018). An ethnobotanical study of medicinal plants in the Greek islands of north aegean region. Front. Pharmacol. 9, 409. 10.3389/fphar.2018.00409 29875656 PMC5974156

[B15] Azizi H. Ghafari S. Ghods R. Shojaii A. Salmanian M. Ghafarzadeh J. (2018). A review study on pharmacological activities, chemical constituents, and traditional uses of *Echium amoenum* . Pharmacognosy Rev. 12 (24), 208–213.

[B16] Barros L. Dueñas M. Carvalho A. M. Ferreira I. C. F. R. Santos-Buelga C. (2012). Characterization of phenolic compounds in flowers of wild medicinal plants from Northeastern Portugal. Food Chem. Toxicol. 50 (5), 1576–1582. 10.1016/j.fct.2012.02.004 22342808

[B17] Başer H. Altintaş A. Kürkçüoglu M. (2013). Turkish rose. A review of the history, ethnobotany, and modern uses of rose petals, rose oil, rose water, and other rose products. Herbalgram 96, 40–53.

[B18] Baser H. Honda G. Miki W. (1986). Herb drugs and herbalists in Turkey. Studia culturae islamicae. Tokyo: Institute for the Study of Languages and Cultures of Asia and Africa.

[B19] Baydoun S. Chalak L. Dalleh H. Arnold N. (2015). Ethnopharmacological survey of medicinal plants used in traditional medicine by the communities of Mount Hermon, Lebanon. J. Ethnopharmacol. 173, 139–156. 10.1016/j.jep.2015.06.052 26165826

[B20] Bellakhdar J. (1997). La pharmacopée marocaine traditionnelle. Médecine arabe ancienne et savoirs populaires. Paris: Ibis Press.

[B21] Bisset N. G. (1994). Herbal Drugs and Phytopharmaceuticals. A handbook for practice on a scientific basis. Stuttgart: Medpharm.

[B22] Boskabady M. H. Shafei M. N. Saberi Z. Amini S. (2011). Pharmacological effects of *Rosa damascena* . Iran J. Basic Med. Sci. 14 (4), 295–307. 23493250 PMC3586833

[B23] Carmona M. D. Llorach R. Obon C. Rivera D. (2005). “Zahraa”, a Unani multicomponent herbal tea widely consumed in Syria: components of drug mixtures and alleged medicinal properties. J. Ethnopharmacology 102, 344–350. 10.1016/j.jep.2005.06.030 16084679

[B25] Crowfoot G. Baldensperger L. (1932). From cedar to hyssop. A study in the folklore of plants in Palestine. London: The Sheldon Press.

[B26] Da-Costa-Rocha I. Bonnlaender B. Sievers H. Pischel I. Heinrich M. (2014). *Hibiscus sabdariffa* L. - a phytochemical and pharmacological review. Food Chem. 165, 424–443. 10.1016/j.foodchem.2014.05.002 25038696

[B27] Davis P. H. (1965). Flora of Turkey and the East Aegean Islands, Vol. 1. Edinburgh: Edinburgh University Press.

[B28] Davis P. H. (1972). Flora of Turkey and the East Aegean Islands, Vol. 4. Edinburgh: Edinburgh University Press.

[B29] Davis P. H. (1982). Flora of Turkey and the East Aegean Islands, Vol. 7. Edinburgh: Edinburgh University Press.

[B30] Deeb T. Knio K. Shinwari Z. K. Kreydiyyeh S. Baydoun E. (2013). Survey of medicinal plants currently used by herbalists in Lebanon. Pak. J. Bot. 45 (2), 543–555.

[B31] Delfan B. Bahmani M. Hassanzadazar H. Saki K. Rafieian-Kopaei M. (2014). Identification of medicinal plants affecting on headaches and migraines in Lorestan Province, West of Iran. Asian Pac. J. Trop. Med. 7, 376–379. 10.1016/s1995-7645(14)60261-3 25312153

[B32] Dimopoulos P. Raus T. Strid A. (2020). Flora of Greece web http://portal.cybertaxonomy.org/flora-greece/content.

[B33] Dötterl S. Vereecken N. J. (2010). The chemical ecology and evolution of bee-flower interactions: a review and perspectivesThe present review is one in the special series of reviews on animal-plant interactions. Can. J. Zool. 88 (7), 668–697. 10.1139/z10-031

[B34] Ellen’s Kitchen (2020). Middle eastern and African spice mixes http://www.ellenskitchen.com/recipebox/arabic/spicemix.html.

[B35] Feinbrun-Dothan N. (1977). Flora Palaestina, Part 3, plates. Jerusalem: The Israel Academy of Sciences and Humanities.

[B36] Feinbrun-Dothan N. (1978). Flora Palaestina, Part 3, text. Jerusalem: The Israel Academy of Sciences and Humanities.

[B37] Feinbrun-Dothan N. (1986a). Flora Palaestina, Part 4, plates. Jerusalem: The Israel Academy of Sciences and Humanities.

[B38] Feinbrun-Dothan N. (1986b). Flora Palaestina, Part 4, text. Jerusalem: The Israel Academy of Sciences and Humanities.

[B39] Fernandes L. Casal S. Pereira J. A. Saraiva J. A. Ramalhosa E. (2017). Edible flowers: a review of the nutritional, antioxidant, antimicrobial properties and effects on human health. J. Food Compost. Anal. 60, 38–50. 10.1016/j.jfca.2017.03.017

[B40] Ferreira S. S. Silva A. M. Nunes F. M. (2020). *Sambucus* nigra L. Fruits and flowers: chemical composition and related bioactivities. Food Rev. Int., 1. 10.1080/87559129.2020.1788578

[B41] Fielding J. Turland N. J. Mathew B. (2005). Flowers of Crete. Kew: Royal Botanic Gardens.

[B24] GBIF (2020). Global biodiversity information facility. https://www.gbif.org, (Accessed February 15, 2020).

[B42] Ghorbani M. Araghi A. Shariatifar N. Mirbaha S. H. Abbasabadi B. M. Samarghandian S. (2020). The toxicity effect of *Echium amoenum* on the liver and kidney of mice. Cddt 17. 10.2174/1570163817666200712170922 32652917

[B43] González I. El-Ouardani F. El-Aallal A. (2010). El que no sepa sonreír que no abra tienda. Marruecos. De zocos, medinas y mercados. Gijón: Ediciones Trea.

[B44] Gras A. Parada M. Rigat M. Vallès J. Garnatje T. (2018). Folk medicinal plant mixtures: establishing a protocol for further studies. J. Ethnopharmacology 214, 244–273. 10.1016/j.jep.2017.12.014 29253612

[B45] Guimarães R. Barros L. Carvalho A. M. Ferreira I. C. F. R. (2011). Infusions and decoctions of mixed herbs used in folk medicine: synergism in antioxidant potential. Phytother. Res. 25, 1209–1214. 10.1002/ptr.3366 21308820

[B46] Güneş S. Savran A. Paksoy M. Koşar M. Çakılcıoğlu U. (2017). Ethnopharmacological survey of medicinal plants in Karaisalı and its surrounding (Adana-Turkey). J. Herbal Med. 8, 68–75.

[B47] Gürdal B. Kültür Ş. (2013). An ethnobotanical study of medicinal plants in Marmaris (Muğla, Turkey). J. Ethnopharmacology 146 (1), 113–126. 10.1016/j.jep.2012.12.012 23261486

[B48] Güzel Y. Güzelşemme M. Miski M. (2015). Ethnobotany of medicinal plants used in Antakya: a multicultural district in Hatay Province of Turkey. J. Ethnopharmacol. 174, 118–152. 10.1016/j.jep.2015.07.042 26239155

[B49] Güzel Y. Güzelşemme M. (2018). Wild plants used as herbal tea in Antakya and Defne provinces of Hatay. Anadolu Ege Tarımsal Araştırma Enstitüsü Dergisi 28 (1), 1–5.

[B50] Guzelmeric E. Ristivojević P. Vovk I. Milojković-Opsenica D. Yesilada E. (2017). Quality assessment of marketed chamomile tea products by a validated HPTLC method combined with multivariate analysis. J. Pharm. Biomed. Anal. 132, 35–45. 10.1016/j.jpba.2016.09.030 27693951

[B51] Hanif M. Mehmood M. H. Ishrat G. Virji S. N. Malik A. Ahmed M. (2019). Pharmacological basis for the medicinal use of *Alcea rosea* in airways disorders and chemical characterization of its fixed oils through GC-MS. Pak J. Pharm. Sci. 32 (5), 2347–2355. 31894065

[B52] Hanlidou E. Karousou R. Kleftoyanni V. Kokkini S. (2004). The herbal market of Thessaloniki (N Greece) and its relation to the ethnobotanical tradition. J. Ethnopharmacology 91 (2-3), 281–299. 10.1016/j.jep.2004.01.007 15120452

[B53] Hayek M. (1996). Encyclopaedia of medicinal plants, Vol. 2. Beyrouth: Librairie du Liban.

[B54] Hayek M. (1997). Encyclopaedia of medicinal plants, Vol. 1. Beyrouth: Librairie du Liban.

[B55] Hayek M. (1998a). Encyclopaedia of medicinal plants, Vol. 3. Beyrouth: Librairie du Liban.

[B56] Hayek M. (1998b). Encyclopaedia of medicinal plants, Vol. 4. Beyrouth: Librairie du Liban.

[B57] Hofrichter J. Krohn M. Schumacher T. Lange C. Feistel B. Walbroel B. (2016). *Sideritis* spp. extracts enhance memory and learning in alzheimer's β-amyloidosis mouse models and aged C57Bl/6 mice. Jad 53 (3), 967–980. 10.3233/jad-160301 27258424 PMC4981905

[B58] Hosseinzadeh H. Nassiri-Asl M. (2013). Avicenna's (ibn sina) the Canon of medicine and saffron (*Crocus sativus*): a review. Phytother. Res. 27 (4), 475–483. 10.1002/ptr.4784 22815242

[B59] Inocencio C. Rivera D. Alcaraz F. Tomás-Barberán F. A. (2000). Flavonoid content of commercial capers ( *Capparis* spinosa, *C. sicula* and *C. orientalis* ) produced in mediterranean countries. Eur. Food Res. Tech. 212 (1), 70–74. 10.1007/s002170000220

[B60] IPNI (2020). International plant names index (IPNI) https://www.ipni.org (Accessed 09 06, 2020).

[B61] Jia W. Gao W.-y. Yan Y.-q. Wang J. Xu Z.-h. Zheng W.-j. (2004). The rediscovery of ancient Chinese herbal formulas. Phytother. Res. 18 (8), 681–686. 10.1002/ptr.1506 15476313

[B62] Jürgens A. Witt T. Gottsberger G. (2003). Flower scent composition in Dianthus and *Saponaria* species (Caryophyllaceae) and its relevance for pollination biology and taxonomy. Biochem. Syst. Ecol. 31 (4), 345–357. 10.1016/s0305-1978(02)00173-4

[B63] Kalivas A. Ganopoulos I. Xanthopoulou A. Chatzopoulou P. Tsaftaris A. Madesis P. (2014). DNA barcode ITS2 coupled with high resolution melting (HRM) analysis for taxonomic identification of *Sideritis* species growing in Greece. Mol. Biol. Rep. 41 (8), 5147–5155. 10.1007/s11033-014-3381-5 24802796

[B64] Karousou R. Bosabalidis A. M. Kokkini S. (1992). *Sideritis syriaca* ssp. *syriaca*: glandular trichome structure and development in relation to systematics. Nordic J. Bot. 12 (1), 31–37. 10.1111/j.1756-1051.1992.tb00198.x

[B65] Kovach W. (2007). Mvsp – a MultiVariate statistical package for windows, ver. 3.1. Pentraeth: Kovach Computing Services.

[B66] Kırmızıbekmez H. Demirci B. Yeşilada E. Başer K. H. C. Demirci F. (2009). Chemical composition and antimicrobial activity of the essential oils of *Lavandula stoechas* L. ssp. *stoechas* growing wild in Turkey. Nat. Product. Commun. 4 (7), 1001–1006. 10.1177/1934578X0900400727 19731612

[B67] Latifian E. Funda Arslanoğlu Ş. (2018). Traditional medicinal plants of Azerbaijan Province of Iran. As 09, 157–170. 10.4236/as.2018.91012

[B68] Latmahalleh D. Niyaki S. Vishekaei M. (2011). Effects of plant density and planting pattern on yield and yield components of Iranian ox-tongue (*Echium amoenum* Fisch and Mey) in North of Iran. J. Med. Plants Res. 5 (6), 932–937.

[B69] Lietava J. (1992). Medicinal plants in a middle paleolithic grave shanidar IV? J. Ethnopharmacology 35 (3), 263–266. 10.1016/0378-8741(92)90023-k 1548898

[B70] Lim T. K. (2014). “ Hibiscus sabdariffa ,” in Edible medicinal and non medicinal plants. Vol 8 flowers. Editor Lim T. K. (Dordrecht: Springer), 324–370.

[B71] Liolios C. C. Graikou K. Skaltsa E. Chinou I. (2010). Dittany of Crete: a botanical and ethnopharmacological review. J. Ethnopharmacol. 131 (2), 229–241. 10.1016/j.jep.2010.06.005 20633631

[B72] Mazal O. (1998). Der wiener dioskurides codex medicus graecus I der Osterreichischen nationalbibliothek, 2 vols. Wien: Akademische Druck- u. Verlagsanstalt.

[B73] Menghini L. Massarelli P. Bruni G. Pagiotti R. (2006). Anti-inflammatory and analgesic effects of *Spartium junceum* L. flower extracts: a preliminary study. J. Med. Food 9 (3), 386–390. 10.1089/jmf.2006.9.386 17004903

[B74] Miller R. Owens S. J. Rørslett B. (2011). Plants and colour: flowers and pollination. Opt. Laser Tech. 43 (2), 282–294. 10.1016/j.optlastec.2008.12.018

[B75] Morris M. M. Frixione N. J. Burkert A. C. Dinsdale E. A. Vannette R. L. (2020). Microbial abundance, composition, and function in nectar are shaped by flower visitor identity. FEMS Microbiol. Ecol. 96 (3), fiaa003. 10.1093/femsec/fiaa003 31922546

[B76] Moshiri M. Vahabzadeh M. Hosseinzadeh H. (2015). Clinical applications of saffron (*Crocus sativus*) and its constituents: a review. Drug Res. (Stuttg) 65 (6), 287–295. 10.1055/s-0034-1375681 Epub 2014 May 21. PMID: 24848002. 24848002

[B77] Moslemi F. Alijaniha F. Naseri M. Kazemnejad A. Charkhkar M. Heidari M. R. (2019). *Citrus aurantium* aroma for anxiety in patients with acute coronary syndrome: a double-blind placebo-controlled trial. J. Altern. Complement. Med. 25 (8), 833–839. 10.1089/acm.2019.0061 31211612

[B78] Mouterde M. (1966). Nouvelle flore du Liban et de la Syrie. Vol. 1. Atlas and Text. Beirut: Dar El- Machreq.

[B79] Mouterde M. (1970). Nouvelle flore du Liban et de la Syrie. Vol. 2. Atlas and Text. Beirut: Dar El- Machreq.

[B80] Mouterde M. (1983). Nouvelle flore du Liban et de la Syrie. Vol. 3. Atlas and Text. Beirut: Dar El- Machreq.

[B81] Nanni V. Canuti L. Gismondi A. Canini A. (2018). Hydroalcoholic extract of *Spartium junceum* L. flowers inhibits growth and melanogenesis in B16-F10 cells by inducing senescence. Phytomedicine 46, 1–10. 10.1016/j.phymed.2018.06.008 30097108

[B82] Nayebi N. Khalili N. Kamalinejad M. Emtiazy M. (2017). A systematic review of the efficacy and safety of *Rosa damascena* Mill. with an overview on its phytopharmacological properties. Complement. Therapies Med. 34, 129–140. 10.1016/j.ctim.2017.08.014 Epub 2017 Aug 25. PMID: 28917365. 28917365

[B83] Ndhlala A. R. Finnie J. F. Van Staden J. (2011). Plant composition, pharmacological properties and mutagenic evaluation of a commercial Zulu herbal mixture: imbiza ephuzwato. J. Ethnopharmacology 133 (2), 663–674. 10.1016/j.jep.2010.10.053 21040765

[B84] Nehmé M. (1980). Fleur sauvages du Liban. Beirut: Conseil National de la Recherche Scientifique.

[B85] Nisrine K. Jihad N. Mariana Y. Noura S. Rabih T. (2016). “Opportunities and limitations in medicinal and aromatic plants’ markets and research in developing countries: Lebanon as a case study,” in Therapeutic medicinal plants, from lab to the market. Editors Teixeira M. Rai M. (Boca Raton: CRC Press), 107–128.

[B86] NYBG (2020a). Universidad de Murcia http://sweetgum.nybg.org/science/ih/herbarium-details/?irn=126299.

[B87] NYBG (2020b). Universidad Miguel Hernández http://sweetgum.nybg.org/science/ih/herbarium-details/?irn=34039.

[B88] Obón C. Rivera D. Alcaraz F. Attieh L. (2014). Beverage and culture. "Zhourat", a multivariate analysis of the globalization of a herbal tea from the Middle East. Appetite 79, 1–10. 10.1016/j.appet.2014.03.024 24703931

[B89] Obón O. Rivera D. (1994). Taxonomic revision of the Section Sideritis (genus Sideritis) (Labiatae). Lehre: J. Cramer.

[B90] Osbaldeston T. Wood R. (2020). Dioscorides de Materia medica. Johannesburg: Ibidis Press.

[B91] Papanikolau K. Kokkini S. (1982). “A taxonomic revision of *Sideritis* L. Section *Empedoclia* (Rafin.) Bentham,” in Aromatic plants: basic and applied aspects. Editors Koedam M. Vokou D. (The Hage: Martinus Nijhoff Publishers), 101–128.

[B92] Papaporfyriou P. K. Sarrou E. Avramidou E. Abraham E. M. (2020). Abundance and phenotypic diversity of the medicinal *Sideritis scardica* Griseb. In relation to floristic composition of its habitat in northern Greece. Sustainability 12 (6), 2542. 10.3390/su12062542

[B93] Patelou E. Chatzopoulou P. Polidoros A. N. Mylona P. V. (2020). Genetic diversity and structure of *Sideritis raeseri* Boiss. & Heldr. (Lamiaceae) wild populations from Balkan Peninsula. J. Appl. Res. Med. Aromatic Plants 16, 100241. 10.1016/j.jarmap.2020.100241

[B94] Perrier X. Flori A. Bonnot F. (2003). “Data analysis methods,” in Genetic diversity of cultivated tropical plants. Editors Hamon P. Seguin M. Perrier X. Glaszmann J. C. (Montpellier: Enfield, Science Publishers), 43–76.

[B95] Perrier X. Jacquemoud-Collet J. P. (2006). Darwin software http://darwin.cirad.fr/(Accessed 10 23, 2020).

[B96] Post G. E. Dinsmore J. E. (1932). Flora of Syria, Palestine and Sinai. Beirut: American Press.

[B97] POWO (2020). Plants of the world online http://www.plantsoftheworldonline.org/(Accessed 09 30, 2020).

[B98] Rabbani M. Sajjadi S. E. Khalili S. (2011). A Lack of tolerance to the anxiolytic action of *Echium amoenum* . Res. Pharm. Sci. 6 (2), 101–106. PMID: 22224093; PMCID: PMC3249772. 22224093 PMC3249772

[B99] Rabbani M. Sajjadi S. E. Vaseghi G. Jafarian A. (2004). Anxiolytic effects of *Echium amoenum* on the elevated plus-maze model of anxiety in mice. Fitoterapia 75 (5), 457–464. 10.1016/j.fitote.2004.04.004 15261383

[B100] Raguso R. A. (2004). “Why do flowers smell? The chemical ecology of fragrance-driven pollination,” in Advances in insect chemical ecology. Editors Cardé R. Millar J. (Cambridge: Cambridge University Press), 151–178.

[B101] Ren D. (1998). Flower-associated Brachycera flies as fossil evidence for Jurassic angiosperm origins. Science 280 (5360), 85–88. 10.1126/science.280.5360.85 9525862

[B102] Rivera D. Obón C. (1995b). The ethnopharmacology of Madeira and Porto Santo islands, a review. J. Ethnopharmacol. 46 (2), 73–93. 10.1016/0378-8741(95)01239-A 7650952

[B103] Rivera D. Allkin R. Obón C. Alcaraz F. Verpoorte R. Heinrich M. (2014). What is in a name? The need for accurate scientific nomenclature for plants. J. Ethnopharmacology 152 (3), 393–402. 10.1016/j.jep.2013.12.022 24374235

[B104] Rivera D. Matilla G. Obón C. Alcaraz F. (2012). Plants and humans in the Near East and the Caucasus: ancient and traditional uses of plants as food and medicine: an ethnobotanical diachronic review:(Armenia, Azerbaijan, Georgia, Iran, Iraq, Lebanon, Syria and Turkey), 1 and 2. Murcia: Editum.

[B105] Rivera D. Obón C. (1995a). Medicinal plants and a multipurpose complex mixture sold in the market of funchal (island of Madeira, Portugal). Ethnobotany 7, 75–82.

[B106] Rivera D. Obón C. (2004). “New functional foods for age-related diseases,” in Functional foods, ageing and degenerative disease. Editors Remacle C. Reusens B. (Boca Raton: CRC Press), 57–80.

[B107] Rivera D. Obón C. Tomás-Lorente F. Ferreres F. Tomás-Barberán F. (1990). Infrasectional systematics of the genus *Sideritis* L. Section *Sideritis* (Lamiaceae). Bot. J. Linn. Soc. 103 (4), 325–349. 10.1111/j.1095-8339.1990.tb00194.x

[B108] Romanucci V. Di Fabio G. D'Alonzo D. Guaragna A. Scapagnini G. Zarrelli A. (2017). Traditional uses, chemical composition and biological activities of *Sideritis raeseri* Boiss. & Heldr. J. Sci. Food Agric. 97 (2), 373–383. 10.1002/jsfa.7867 27342219

[B109] Rouhi-Boroujeni H. Asadi-Samani M. Moradi M. (2016). A review of the medicinal plants effective on headache based on the ethnobotanical documents of Iran. Der Pharmacia Lettre 8 (3), 37–42.

[B110] Sadeghi L. Tanwir F. Yousefi Babadi V. (2018a). Physiological and biochemical effects of *Echium amoenum* extract on Mn2+-imposed Parkinson like disorder in rats. Adv. Pharm. Bull. 8 (4), 705–713. 10.15171/apb.2018.079 30607343 PMC6311646

[B111] Sadeghi L. Yousefi Babadi V. Tanwir F. (2018b). Improving effects of *Echium amoenum* aqueous extract on rat model of Alzheimer's disease. Jin 17 (3-4), 661–669. 10.3233/JIN-180093 PMID: 30103344. 30103344

[B112] Saeed I. A. Ali L. Jabeen A. Khasawneh M. Rizvi T. A. Ashraf S. S. (2013). Estrogenic activities of ten medicinal herbs from the Middle East. J. Chromatogr. Sci. 51 (1), 33–39. 10.1093/chromsci/bms101 22700791

[B113] Sahin F. P. Duman H. Ezer N. (2008). Comparative morphological investigation of *Sideritis* species II: *S. cilicic*a. Boiss. & Bal. & S. niveotomentosa Hub.-Mor. Turk J. Pharm. Sci. 5 (1), 35–44. Available at: http://cms.galenos.com.tr/Uploads/Article_12538/35-45.pdf

[B114] Saito N. Harborne J. B. (1992). Correlations between anthocyanin type, pollinator and flower colour in the Labiatae. Phytochemistry 31 (9), 3009–3015. 10.1016/0031-9422(92)83437-4

[B115] Salvador A. C. Guilherme R. J. R. Silvestre A. J. D. Rocha S. M. (2019). “ *Sambucus* nigra berries and flowers health benefits: from lab testing to human consumption,” in Bioactive molecules in food, reference series in phytochemistry. Editors Mérillon J.-M. Ramawat K. G. (Switzerland: Springer Natura). 10.1007/978-3-319-78030-6_46 Chapter 77.

[B116] Sargin S. A. Selvi S. López V. (2015). Ethnomedicinal plants of sarigöl district (Manisa), Turkey. J. Ethnopharmacol. 171, 64–84. 10.1016/j.jep.2015.05.031 26026370

[B117] Sarris J. Panossian A. Schweitzer I. Stough C. Scholey A. (2011). Herbal medicine for depression, anxiety and insomnia: a review of psychopharmacology and clinical evidence. Eur. Neuropsychopharmacol. 21 (12), 841–860. 10.1016/j.euroneuro.2011.04.002 21601431

[B118] Sayyah M. Boostani H. Pakseresht S. Malaieri A. (2009). Efficacy of aqueous extract of *Echium amoenum* in treatment of obsessive-compulsive disorder. Prog. Neuro-Psychopharmacology Biol. Psychiatry 33 (8), 1513–1516. 10.1016/j.pnpbp.2009.08.021 19737592

[B119] Sidor A. Gramza-Michałowska A. (2015). Advanced research on the antioxidant and health benefit of elderberry (*Sambucus nigra*) in food - a review. J. Funct. Foods 18, 941–958. 10.1016/j.jff.2014.07.012

[B120] Singh O. Khanam Z. Misra N. Srivastava M. (2011). Chamomile (*Matricaria chamomilla* L.): an overview. Phcog Rev. 5 (9), 82–95. 10.4103/0973-7847.79103 22096322 PMC3210003

[B121] Smigielski K. Prusinowska R. Stobiecka A. Kunicka-Styczyñska A. Gruska R. (2018). Biological properties and chemical composition of essential oils from flowers and aerial parts of lavender (*Lavandula angustifolia*). J. Essent. Oil Bearing Plants 21 (5), 1303–1314. 10.1080/0972060x.2018.1503068

[B122] Sotiropoulou N. S. Megremi S. F. Tarantilis P. (2020). Evaluation of antioxidant activity, toxicity, and phenolic profile of aqueous extracts of chamomile (*Matricaria chamomilla* L.) and sage (*Salvia officinalis* L.) prepared at different temperatures. Appl. Sci. 10 (7), 2270. 10.3390/app10072270

[B123] Srivastava R. Ahmed H. Dixit R. Dharamveer S. Saraf S. (2010). *Crocus sativus* L.: a comprehensive review. Phcog Rev. 4 (8), 200–208. 10.4103/0973-7847.70919 22228962 PMC3249922

[B124] Stanoeva J. P. Stefova M. Stefkov G. Kulevanova S. Alipieva K. Bankova V. (2015). Chemotaxonomic contribution to the *Sideritis* species dilemma on the Balkans. Biochem. Syst. Ecol. 61, 477–487. 10.1016/j.bse.2015.07.008

[B125] Statista (2020). Saffron production worldwide in 2019, by leading country https://www.statista.com/statistics/1135621/leading-saffron-producers-worldwide/.

[B126] Tadić V. M. Jeremic I. Dobric S. Isakovic A. Markovic I. Trajkovic V. (2012). Anti-inflammatory, gastroprotective, and cytotoxic effects of *Sideritis scardica* extracts. Planta Med. 78 (05), 415–427. 10.1055/s-0031-1298172 22274814

[B127] Todorova M. Trendafilova A. (2014). *Sideritis scardica* Griseb., an endemic species of Balkan peninsula: traditional uses, cultivation, chemical composition, biological activity. J. Ethnopharmacol. 152 (2), 256–265. 10.1016/j.jep.2014.01.022 24487281

[B128] Tohme G. Tohme H. (2007). Illustrated flora of Lebanon. Beirut: National Council for Scientific Research.

[B129] TPL (2020). The plant list http://www.theplantlist.org/(Accessed 10 23, 2020).

[B130] Tschiggerl C. Bucar F. (2012). Guaianolides and volatile compounds in chamomile tea. Plant Foods Hum. Nutr. 67 (2), 129–135. 10.1007/s11130-012-0277-1 22410959

[B131] Tsioutsiou E. E. Giordani P. Hanlidou E. Biagi M. De Feo V. Cornara L. (2019). Ethnobotanical study of medicinal plants used in Central Macedonia, Greece. Evid. Based Complement. Alternat Med. 2019, 1. 10.1155/2019/4513792 PMC6463668 31057648

[B132] Université Saint-Joseph de Beyrouth (2009). Flore virtuelle du Liban http://fs.usj.edu.lb/flore_du_liban/index.php (Accessed October 8, 2012).

[B133] Varsani M. Graikou K. Velegraki A. Chinou I. (2017). Phytochemical analysis and antimicrobial activity of *Origanum dictamnus* traditional herbal tea (decoction). Nat. Product. Commun. 12 (11), 1801–1804. 10.1177/1934578x1701201139

[B134] Vasilopoulou C. G. Kontogianni V. G. Linardaki Z. I. Iatrou G. Lamari F. N. Nerantzaki A. A. (2013). Phytochemical composition of “mountain tea” from *Sideritis clandestina* subsp. *clandestina* and evaluation of its behavioral and oxidant/antioxidant effects on adult mice. Eur. J. Nutr. 52 (1), 107–116. 10.1007/s00394-011-0292-2 22202940

[B135] Vinokur Y. Rodov V. Reznick N. Goldman G. Horev B. Umiel N. (2006). Rose petal tea as an antioxidant‐rich beverage: cultivar effects. J. Food Sci. 71 (1), 42–47. 10.1111/j.1365-2621.2006.tb12404.x

[B136] Wichtl M. (1984). Teedrogen. Ein handbuch für apotheker und Ärzte. Stuttgart: Wissenschaftliche Wissenschaftliche.

[B137] Wiersema J. Schori M. (2020). Query all GRIN-global taxonomy for plants https://npgsweb.ars-grin.gov/gringlobal/taxon/taxonomysearch.aspx (Accessed 09 03, 2020).

[B138] Yu F. Takahashi T. Moriya J. Kawaura K. Yamakawa J. Kusaka K. (2006). Traditional Chinese medicine and Kampo: a review from the distant past for the future. J. Int. Med. Res. 34 (3), 231–239. 10.1177/147323000603400301 16866016

[B139] Zohary M. (1966a). Flora Palaestina, Part 1, plates. Jerusalem: The Israel Academy of Sciences and Humanities.

[B140] Zohary M. (1966b). Flora Palaestina, Part 1, text. Jerusalem: The Israel Academy of Sciences and Humanities.

[B141] Zohary M. (1972a). Flora Palaestina, Part 2, plates. Jerusalem: The Israel Academy of Sciences and Humanities.

[B142] Zohary M. (1972b). Flora Palaestina, Part 2, text. Jerusalem: The Israel Academy of Sciences and Humanities.

[B143] Zurayk R. Talhouk S. (2009). Plants and people. Ethnobotanical knowledge from Lebanon. Beirut, ibsar and slow food.

