# Supplementary material for: A Comparison Study on Traditional Mixtures of Herbal Teas Used in Eastern Mediterranean Area
Source: Front Pharmacol. 2021 Apr 23;12:632692. doi: 10.3389/fphar.2021.632692 (PMC8103161; doi:10.3389/fphar.2021.632692)
Supplement: Supplementary file 2 [file table1.docx]

### Supplementary Table 1

Abbreviations: Ing.: number of ingredients. Medicinal uses reported by our informants or in the labels, in order of increasing frequency: CIR, Circulatory; CR, Anticancer; HA, Headache; DEP, Depurative; TO, Tonic; CO, Treatment of common cold; GAF, has a good aroma and flavor; APH, Aphrodisiac; SD, Sedative; HM, Good for health, herbal teas, consumed to preserve health and prevent diseases; DIG, Digestive, good for stomach.

| Voucher | Type | Source | Code analyses | Locality origin | Region | Country | Ref date | Medicinal Uses | Name | Weight (g) | Batch number | Ing. | Herbaria |
| --- | --- | --- | --- | --- | --- | --- | --- | --- | --- | --- | --- | --- | --- |
| TUR_01 | Mix | Eksim domestic and foreign trade (Eksim iç ve diç Ticaret) | 001/2019 | Eminönü | Istanbul | Turkey | 2001 | GAF | Alaeddin Turkish Apple Tea | 125 | ? | 6 | UMH |
| GR_002 | Simple | Aroma Physis - Bioagros AE | 002/2019 | Kria Brise / Cold Fountain, Pella | Macedonia | Greece | 05/04/2018 | HM, DIG | Mountain TEA - Tschai Bounoy scardica - ΤΣΑΙ ΒΟΥΝΟΥ | 20 | 515311218 | 1 | UMH |
| GR_003 | Simple | TRADITIONAL CONFECTIONERY CENTRAL (ΠΑΡΑΔΟΣΙΑΚΟ ΖΑΧΑΡΟΠΛΑΣΤΕΙΟ ΚΕΝΤΡΙΚΟΝ) | 003/2020 | Litóchoro | Pieria | Greece | ? | HM, DIG | MOUNTAIN TEA OLYMPUS - ΤΣΑΙ ΒΟΥΝΟΥ ΟΛΥΜΠΟΥ | 70 | ? | 1 | UMH |
| GR_004 | Simple | TRADITIONAL CONFECTIONERY CENTRAL (ΠΑΡΑΔΟΣΙΑΚΟ ΖΑΧΑΡΟΠΛΑΣΤΕΙΟ ΚΕΝΤΡΙΚΟΝ) | 004/2020 | Litóchoro | Olympus | Greece | 2019 | HM, DIG | MOUNTAIN TEA OLYMPUS - ΤΣΑΙ ΒΟΥΝΟΥ ΟΛΥΜΠΟΥ | ? | ? | 1 | UMH |
| GR_005 | Simple | ? | 005/2020 | ? | Eubea | Greece | 01/12/2016 | HM, DIG | MOUNTAIN-EVIA TEA - ΤΣΑΙ ΒΟΥΝΟΥ-ΕΥΒΟΙΑΣ | 70 | 42705 | 1 | UMH |
| GR_006 | Simple | ? | 006/2020 | Olympus | Macedonia | Greece | 19/04/2017 | HM, DIG | MOUNTAIN TEA OLYMPUS - ΤΣΑΙ ΒΟΥΝΟΥ ΟΛΥΜΠΟΥ | 50 | 42844 | 1 | UMH |
| GR_007 | Simple | ? | 007/2020 | ? | ? | Greece | ? | HM, DIG | MOUNTAIN ELCHYMA TEA - ΤΣΑΙ ΒΟΥΝΟΥ ΕΛΛΧΥΜΑ | 48 | ? | 1 | UMH |
| GR_008 | Simple | Elixir ( Herbs, oils, spices and teas) | 008/2020 | Collected on the slopes of Dirfis (Dirphys), (the great mountain of Euboea) | Eubea | Greece | ? | HM, DIG | Blossom Tea Leaves Self - ΤΣΑΙ ΑΝΘΟΣ ΦΥΛΛΑ ΑΥΤΟΦΥΗ | 50 | ? | 1 | UMH |
| GR_009 | Simple | Mountain Herbal Tea (Bergkräutertee) | 009/2020 | Volos | Tesalia | Greece | ? | HM, DIG | MOUNTAIN TEA Volos - ΤΣΑΙ ΒΟΥΝΟΥ Βολος | 100 | 0716TB5GR1 | 1 | UMH |
| GR_010 | Simple | Mountain Herbal Tea (Bergkräutertee) | 010/2020 | Volos | Tesalia | Greece | ? | HM, DIG | MOUNTAIN TEA Volos - ΤΣΑΙ ΒΟΥΝΟΥ Βολος | 100 | ΤΣΒ3061581 | 1 | UMH |
| GR_011 | Simple | ? | 011/2020 | Olympus | Macedonia | Greece | ? | HM, DIG | ? - ? | ? | ? | 1 | UMH |
| GR_012 | Simple | ? | 012/2020 | ? | ? | Greece | 2018 | HM, DIG | ? - ? | ? | ? | 1 | UMH |
| GR_013 | Simple | ? | 013/2020 | ? | ? | Greece | ? | HM, DIG | MOUNTAIN TEA - ΤΣΑΙ ΒΟΥΝΟΥ | ? | ? | 1 | UMH |
| GR_014 | Simple | Herbs and spices collection, E. Kabrianis Family | 014/2020 | ? | Crete | Greece | ? | HM, DIG | Malotira - Μαλοτίρα | 20 | ? | 1 | UMH |
| GR_015 | Simple | Brothers Ympitsiouni (Αφοι υμπιτσιουνη ο.ε.) | 015/2020 | ? | ? | Greece | ? | HM, DIG | MOUNTAIN TEA - ΤΣΑΙ ΤΟΥ ΒΟΥΝΟΥ | ? | ? | 1 | UMH |
| GR_016 | Simple | PAPOULIAS CHR.- MILIONI AN. & CΙΑ Ε.Ε (ΠΑΠΟΥΛΙΑΣ ΧΡ.- ΜΗΛΙΩΝΗ ΑΝ. & ΣΙΑ Ε.Ε) | 016/2020 | Arcadia | Peloponeso | Greece | ? | HM, DIG | Mountain Tea - Τσαι Βουνου | 60 | ? | 1 | UMH |
| GR_017 | Simple | TASTE & HEALTH (ΓΕΥΣΗ & ΥΓΕΙΑ) | 017/2020 | Eubea | Athens | Greece | 2018 | HM, DIG | MOUNTAIN - EVIA TEA - ΤΣΑΙ ΒΟΥΝΟΥ - ΕΥΒΟΙΑΣ | ¿60? | ? | 1 | UMH |
| GR_018 | Simple | TASTE & HEALTH (ΓΕΥΣΗ & ΥΓΕΙΑ) | 018/2020 | Volos | Athens | Greece | 2018 | HM, DIG | MOUNTAIN TEA - PELION - ΤΣΑΙ ΒΟΥΝΟΥ - ΠΗΛΙΟ | ¿60? | ? | 1 | UMH |
| GR_019 | Simple | TASTE & HEALTH (ΓΕΥΣΗ & ΥΓΕΙΑ) | 019/2020 | Olympus | Athens | Greece | 2018 | HM, DIG | MOUNTAIN - OLYMPUS TEA - ΤΣΑΙ ΒΟΥΝΟΥ - ΟΛΥΜΠΟΥ | 60 | ? | 1 | UMH |
| GR_020 A | Mix | K.A TZIMPLAKIS | 020/2020 (A) | Naxos | Naxos | Greece | 2018 | HM | ? - ? | ? | ? | 36 | UMH |
| GR_020 B | Mix | K.A TZIMPLAKIS | 020/2020 (B) | Naxos | Naxos | Greece | 2018 | HM | ? - ? | ? | ? | 38 | UMH |
| GR_021 | Mix | Barbakeion Market | 021/2020 | Athens | Ática | Greece | 2018 | HM | TEA OF FLOWERS - ΤΣΑΓΙ ΑΡΩΜΑΤΩΝ | ? | ? | 8 | UMH |
| GR_022 | Mix | AGRECOFARMS, HELLENIC GOODS HOUSE (ΟΙΚΟΣ ΕΛΛΗΝΙΚΩΝ ΑΓΑΘΩΝ) | 022/2020 | Heraklion | Crete | Greece | ? | HM | Cretan 40 herbs Sarantavotano (Organic Herbs) - Κρητικά 40 βότανα Σαρανταβότανο (Βιολογικα Βοτανα) | 60 | ? | 25 | UMH |
| GR_023 | Simple | AGRECOFARMS, HELLENIC GOODS HOUSE (ΟΙΚΟΣ ΕΛΛΗΝΙΚΩΝ ΑΓΑΘΩΝ) | 023/2020 | Heraklion | Crete | Greece | ? | HM | Cretan dictamus herbal infusion (Organic Herbs) - Κρητικό dictamus φυτική έγχυση (Βιολογικα Βοτανα) | 60 | ? | 1 | UMH |
| IR_024 | Mix | Green desert drink (SABZ NOSHE KAVIR) | 024/2020 | Tehran | Central District | Iran | 2015 | HM | Dr. Bean Herbal Tea - ¿? | 75 | SDI 04.15 | 12 | UMH |
| GR_025 | Mix | BOTANA OLYMPUS / OLYMPUS SENSES | 025/2020 | Thera | Santorini | Greece | 2018 | CO | Olympus Mix of Herbs for Immune system - Μίγμα βοτάνων Ολυμπός | 100 |  | 15 | UMH |
| GR_026 | Mix | BOTANA OLYMPUS / OLYMPUS SENSES | 026/2020 | Thera | Santorini | Greece | 2018 | CO | Respiratory and Cough Herb Mix - μείγμα βότανον Αναπνευστικό και βήχα | 100 |  | 6 | UMH |
| GR_027 | Mix | EGGIN | 027/2020 | Rethymno | Crete | Greece | 13/04/2017 | HM | Mix Tea - τσαι αναμεικ το | 50 | TSA5117 | 8 | UMH |
| GR_028 | Mix | MOKA STORE NATURAL PRODUCTS | 028/2020 | Rethymno | Crete | Greece | 13/04/2017 | HM | Mixed Tea (Cretan Varieties) - σαρανταδεντρι (σορόκοτραβιε) | 90 | ? | 6 | UMH |
| GR_029 | Mix | Móka | 029/2020 | Rethymno | Crete | Greece | 13/04/2017 | HM | 40 tree, Cretan herbs cocktail - 40 δεντρί, κοκτέιλ κρητικά βότανα | 56 | ? | 7 | UMH |
| GR_030 | Mix | AMARI ANEA | 030/2020 | Rethymno | Crete | Greece | 13/04/2017 | HM | Drink Mixed - ρόφημα ανάμικτο | 30 | 185713 | 11 | UMH |
| GR_031 | Mix | CRETAN FARMERS | 031/2020 | Rethymno | Crete | Greece | 13/04/2017 | CO | Forty, Karteraki, herbal mixture for colds - Σαρανταδéntri, Κάρτερáκι, μειγμα βοτανων για το κρυολογημα | 30 | LS0816 | 8 | UMH |
| GR_032 | Mix | FOTSI | 032/2020 | Athens | Athens | Greece | 01/04/2017 | HM, APH | from 1930 …, spices Fotsi- απó το 1930…, μπαχαρικά, Φώτση | 30 | ? | 11 | UMH |
| GR_033 | Mix | Minoan Life Herbs and Spices | 033/2020 | Rethymno | Crete | Greece | 01/04/2017 | DIG, CO, HA | Herbs mixture (stomachache, headache, flu, cold) - μείγμα βότανον | 20 | 17180332 | 5 | UMH |
| GR_034 | Mix | Cretan Beauty and Co. | 034/2020 | Heraklion | Crete | Greece | 01/04/2017 | HM | Cretan Mix Tea - Κρητικό ανάμεικτο τσάι | 120 | L06717 | 7 | UMH |
| GR_035 | Mix | AGRECOFARMS, HELLENIC GOODS HOUSE (ΟΙΚΟΣ ΕΛΛΗΝΙΚΩΝ ΑΓΑΘΩΝ) | 035/2020 | Heraklion | Crete | Greece | 01/04/2017 | HM | Organic Herbs Mixed Tea - Μικτό τσάι οργανικών βοτάνων | 20 | 0300003 10117 | 7 | UMH |
| GR_036 | Mix | KARTERAKI | 036/2020 | Heraklion | Crete | Greece | 01/04/2017 | HM | Mix tea - Ανáμεικτό τσáη | 50 | TSM32316 | 8 | UMH |
| GR_037 | Mix | MARAVEL HERBS | 037/2020 | Rethymno | Crete | Greece | 01/04/2017 | HM | Cretan Mountain Herbs - Κρητικά ορεινά βότανα | ? | ? | 6 | UMH |
| GR_038 | Mix | Taste & Health, Herbs and Spices from the world (Γεύση & Υγεία, Βóτάνα και Μπαχαρικά από τον κóσμό) | 038/2020 | Athens | Athens | Greece | 01/04/2017 | SD | Good Sleep | 40 | Φ Φ-1458 | 10 | UMH |
| ZH_S1_LEB | Mix | S 1 (g) | S 1 (g)/2011 | Kfarhaman | South Lebanon | Lebanon | 15/11/2011 | HM. DIG, SD | Zhourat | 250 | ? | 24 | MUB |
| ZH_S2_LEB | Mix | S 2 (g) | S 2 (g)/2011 | Kfarhaman | South Lebanon | Lebanon | 15/11/2011 | HM. DIG, SD | Zhourat | 250 | ? | 33 | MUB |
| ZH_S3_LEB | Mix | S 3 (g) | S 3 (g)/2011 | Kfarhaman | South Lebanon | Lebanon | 15/11/2011 | HM. DIG, SD | Zhourat | 250 | ? | 22 | MUB |
| ZH_TR_LEB | Mix | Tripoli | Tripoli/2011 | Tripoli | North Lebanon | Lebanon | 15/11/2011 | HM | Zhourat | 250 | ? | 11 | MUB |
| ZH_PA_LEB1 | Mix | Paris 1 | Paris 1/2011 | Beirut | Beirut | Lebanon | 15/11/2011 | HM | Zhourat | 250 | ? | 11 | MUB |
| ZH_PA_LEB2 | Mix | Paris 2 | Paris 2/2011 | Beirut | Beirut | Lebanon | 15/11/2011 | HM | Zhourat | 250 | ? | 13 | MUB |
| ZH_A_SYR | Mix | Damascus Central Market | A/2003 | Damascus | Damascus | Syria | 14/09/1999 | HM; SD | True Damascus’ Flowers Tea | 200 | ? | 12 | MUB |
| ZH_B_SYR | Mix | Damascus Central Market | B/2003 | Damascus | Damascus | Syria | 09/09/1999 | HM; SD | Flowers Tea | 200 | ? | 15 | MUB |
| ZH_C_SYR | Mix | Damascus Central Market | C/2003 | Damascus | Damascus | Syria | 09/09/1999 | HM; SD | Zhourat | 200 | ? | 6 | MUB |
| ZH_D_SYR | Mix | Drug Store Chez Olabi, Damascus Central Market | D/2003 | Damascus | Damascus | Syria | 13/11/1999 | HM; SD | Drug Store Chez Olabi | 200 | ? | 8 | MUB |
| ZH_E_SYR | Mix | Damascus Central Market | E/2003 | Damascus | Damascus | Syria | 13/11/1999 | HM; SD | Zhourat | 200 | ? | 7 | MUB |
| ZH_F_SYR | Mix | Drug Store El Tabbaa brothers (Damascus Central Market) | F/2003 | Damascus | Damascus | Syria | 13/11/1999 | HM; SD | Drug Store El Tabbaa brothers | 200 | ? | 8 | MUB |
| ZH_LT_LEB | Mix | London Tea (Lebanon) | 15/2011 | Beirut | Beirut | Lebanon | 15/11/2011 | HM | London Tea (Lebanon) | 100 | ? | 9 | MUB |
| ZH_AS_TUR | Mix | Assyrian Tur-Abdin (Abdalla 2004) | Abdalla 2004 | n/a | n/a | Turkey | n/a | HM | n/a | n/a | n/a | 10 | n/a |
| ZH_SA_SYR | Mix | Zhourat Samia (Syria) | 16/2011 | Damascus | Damascus | Syria | 15/11/2011 | HM | Zhourat Samia (Syria) | 100 | ? | 6 | MUB |
| ZH_ZHO_SYR | Mix | Syrian Natural Products Zhourat tea | 17/2011 | Damascus | Damascus | Syria | 15/11/2011 | HM | Syrian Natural Products Zhourat tea | 100 | ? | 5 | MUB |
| ZH_ALA_SYR | Mix | Alattar | 18/2011 | Damascus | Damascus | Syria | 15/11/2011 | HM | Alattar Zhourat Lebnania (Syria) | 100 | ? | 7 | MUB |
| ZH_ALR_SYR | Mix | Al Rayan | 19/2011 | Damascus | Damascus | Syria | 15/11/2011 | HM | Al Rayan Zhourat | 100 | ? | 5 | MUB |
| ZH_ABI_LEB | Mix | Abido Co. | 20/2011 | Beirut | Beirut | Lebanon | 15/11/2011 | HM | Abido Libanesischer Kräutertee | 100 | ? | 8 | MUB |
| ZH_ANIM_LEB | Mix | Abido Co. | 21/2011 | Beirut | Beirut | Lebanon | 15/11/2011 | HM | Abido Mills Herbal Tea | 100 | ? | 4 | MUB |
| SPI_ARS_SAR | Mix | Arabian Spice (Ellenskitchen.com) | Ellenskitchen.com | n/a | n/a | n/a | n/a | SPI | n/a | n/a | n/a | 13 | n/a |
| SPI_BAH_SAR | Mix | Bahrat (Ellenskitchen.com) | Ellenskitchen.com | n/a | n/a | n/a | n/a | SPI | n/a | n/a | n/a | 8 | n/a |
| SPI_HAW_YEM | Mix | Hawayil (Yemen) (Ellenskitchen.com) | Ellenskitchen.com | n/a | n/a | n/a | n/a | SPI | n/a | n/a | n/a | 5 | n/a |
| SPI_VER_TUN | Mix | Berebere (Ellenskitchen.com) | Ellenskitchen.com | n/a | n/a | n/a | n/a | SPI | n/a | n/a | n/a | 11 | n/a |
| SPI_ZAH_LEB | Mix | Zahtar (Ellenskitchen.com) | Ellenskitchen.com | n/a | n/a | n/a | n/a | SPI | n/a | n/a | n/a | 0 | n/a |
| SPI_LAE_MOR | Mix | Laebriq (Morocco) (Gonz 2010) | Gonz 2010 | n/a | n/a | Morocco | n/a | SPI | n/a | n/a | n/a | 20 | n/a |
| SPI_RAS_MOR | Mix | Ras el Hanut (Morocco) (Gonz 2010) | Gonz 2010 | n/a | n/a | Morocco | n/a | SPI | n/a | n/a | n/a | 22 | n/a |
| SPI_TJA_MOR | Mix | Tjalaet (Morocco) (Gonz 2010) | Gonz 2010 | n/a | n/a | Morocco | n/a | SPI | n/a | n/a | n/a | 12 | n/a |
| SPI_RASB_MOR | Mix | Ras el Hanout (Belladkhdar 1997) | Belladkhdar 1997 | n/a | n/a | Morocco | n/a | SPI | n/a | n/a | n/a | 30 | n/a |
| SPI_ARB_PAL | Mix | Arba'in (Crowfoot & Baldensperger 1932) | Crowfoot & Baldensperger 1932 | n/a | n/a | Palestine | n/a | SPI | n/a | n/a | n/a | 40 | n/a |
| TUR_TSHT | Mix | Grand Turkish Bazaar | 039/2020 | Istanbul | Istanbul | Turkey | 01/06/2020 | SD, CO, GAF, DEP | Turkish Sultan Herbal tea mix | 100 | ? | 5 | UMH |
| TUR_TWTM | Mix | Grand Turkish Bazaar | 040/2020 | Istanbul | Istanbul | Turkey | 01/06/2020 | SD, CO | Turkish winter tea mix | 250 | ? | 9 | UMH |
| TUR_TETM | Mix | Grand Turkish Bazaar | 041/2020 | Istanbul | Istanbul | Turkey | 01/06/2020 | SD, GAF | Turkish energy tea mix | 100 | ? | 7 | UMH |
| TUR_TNFT | Mix | Grand Turkish Bazaar | 042/2020 | Istanbul | Istanbul | Turkey | 01/06/2020 | DIG, DEP, GAF | Turkish herbal form tea | 100 | ? | 11 | UMH |
| TUR_TLTM | Mix | Grand Turkish Bazaar | 043/2020 | Istanbul | Istanbul | Turkey | 01/06/2020 | APH | Turkish love tea mix | 250 | ? | 6 | UMH |
| TUR_TBSG | Mix | Turk Asci Haberleri | 044/2020 | n/a | n/a | Turkey | 01/06/2020 | HM, CO | n/a | n/a | n/a | 8 | n/a |
| TUR_WTHM | Mix | SS Food Wholesale Collective | 045/2020 | Tekirdag | Tekirdag | Turkey | 01/06/2020 | HM, CO | Winter tea herbal mix | 200 | ? | 10 | UMH |
| TUR_GSMN | Mix | kuklisvar.com | 046/2020 |  |  | Turkey | 01/06/2020 | HM, CO, TO | Ottoman tea |  | ? | 11 | UMH |
| IR_IDMHT | Mix | Koolleh | 047/2020 | Tehran | Central District | Iran | 14/08/2020 | HM | Persian dried mixed herbal tea | 100 | ? | 11 | UMH |
| IR_IPRB | Mix | Attari traditional herbsop | 048/2020 | Tehran | Central District | Iran | 14/08/2020 | HM | Mix with rose petals and Echium amoenum flowers | 150 | ? | 8 | UMH |
| IR_ICHRO | Mix | Rostani | 049/2020 | Tehran | Central District | Iran | 14/08/2020 | SD, DIG | Chamomile Rostani mix, 20 silk tea bags | 30 | ? | 2 | UMH |
| IR_IEVE | Mix | Vegafolk | 050/2020 |  |  | Iran | 14/08/2020 | HM | Echium mix, 14 silk tea bags | 28 | 38/14498? | 3 | UMH |
| IR_IEIF | Mix | Pilar | 051/2020 |  |  | Iran | 14/08/2020 | GAF | Eight fruits tea | 300 | 45/12998? | 8 | UMH |
| IR_IHMH | Mix | Golbarg Zarrin | 052/2020 |  |  | Iran | 14/08/2020 | GAF | Hamdam mixed herbal tea, 20 tea bags | 40 | 154822? | 5 | UMH |
| IR_IMMR | Mix | Rostani | 053/2020 | Tehran | Central District | Iran | 14/08/2020 | DIG, TO, CIR | Mint Rostani mix, 20 silk tea bags | 30 | ? | 3 | UMH |
| IR_INBL | Mix | Newsha (Newshadarian Foodstuffs Co) | 054/2020 | Tehran | Central District | Iran | 14/08/2020 | SD, DIG, CIR, HM | Newsha borage, lemon verbena, linden and valerian infusion, 20 tea bags | 40 | 44/133974? | 4 | UMH |
| IR_IMGO | Mix | Golestan | 055/2020 |  |  | Iran | 14/08/2020 | CO, HM | Golestan Mied Herbal Infusion, 20 tea bags | 40 | 16/23306? | 4 | UMH |
| IR_IGOK | Mix | Golkooh (Binalood Gol Kooh) | 056/2020 | Mashhad | Khorasan | Iran | 14/08/2020 | HM | Golkooh mixed herbal tea, 20 tea bags | 40 | 50/13492? | 17 | UMH |
| IR_IREI | Mix | Benli | 057/2020 |  |  | Iran | 14/08/2020 | GAF | Benli reception infusion, 14 silk tea bags | 28 | 60/11038? | 6 | UMH |
| IR_IRCA | Mix | Florist | 058/2020 |  |  | Iran | 14/08/2020 | DEP, CIR, HM, DIG, CR | Red carpet herbal tea in can | 100 | 36/1168? | 4 | UMH |
| IR_ISAC | Mix | Tarvand Saffron Co. | 059/2020 | Ghaen | Khorasan | Iran | 14/08/2020 | SD, CO, CR, HA | Saffron, cardamom and rose tea, 14 silk tea bags | 28 | 56/3985? | 3 | UMH |
| IR_ISHT | Mix | Saharkhiz | 060/2020 | Mashhad | Khorasan | Iran | 14/08/2020 | SD, CO, CR | Saffron herbal tea, 12 pyramid silk tea bags | 24 | 50/19266? | 3 | UMH |
| IR_ITCA | Mix | Newsha (Newshadarian Foodstuffs Co) | 061/2020 | Tehran | Central District | Iran | 14/08/2020 | DIG, HM | Thyme, cumin, ajwain tea, 12 pyramid silk tea bags | 24 | 44/133974? | 3 | UMH |
| IR_IEMH | Mix | Padideh Sabz Mehr-e-Giah Corp. | 062/2020 | Tehran | Central District | Iran | 14/08/2020 | SD, CO, TO, HM | Echium mix herbal tea, 14 pyramid silk tea bags | 28 | ? | 5 | UMH |
